# The effect of the pathological V72I, D109N and T190M missense mutations on the molecular structure of α-dystroglycan

**DOI:** 10.1371/journal.pone.0186110

**Published:** 2017-10-16

**Authors:** Sonia Covaceuszach, Manuela Bozzi, Maria Giulia Bigotti, Francesca Sciandra, Petr V. Konarev, Andrea Brancaccio, Alberto Cassetta

**Affiliations:** 1 Istituto di Cristallografia–CNR, Trieste Outstation, Trieste, Italy; 2 Istituto di Biochimica e Biochimica Clinica, Università Cattolica del Sacro Cuore, Roma, Italy; 3 Istituto di Chimica del Riconoscimento Molecolare—CNR c/o Università Cattolica del Sacro Cuore, Roma, Italy; 4 School of Biochemistry, University of Bristol, Bristol, United Kingdom; 5 A.V. Shubnikov Institute of Crystallography of Federal Scientific Research Centre “Crystallography and Photonics” of Russian Academy of Sciences, Moscow, Russia; 6 National Research Centre “Kurchatov Institute”, Moscow, Russia; University of Lincoln, UNITED KINGDOM

## Abstract

Dystroglycan (DG) is a highly glycosylated protein complex that links the cytoskeleton with the extracellular matrix, mediating fundamental physiological functions such as mechanical stability of tissues, matrix organization and cell polarity. A crucial role in the glycosylation of the DG α subunit is played by its own N-terminal region that is required by the glycosyltransferase LARGE. Alteration in this O-glycosylation deeply impairs the high affinity binding to other extracellular matrix proteins such as laminins. Recently, three missense mutations in the gene encoding DG, mapped in the α-DG N-terminal region, were found to be responsible for hypoglycosylated states, causing congenital diseases of different severity referred as primary dystroglycanopaties.To gain insight on the molecular basis of these disorders, we investigated the crystallographic and solution structures of these pathological point mutants, namely V72I, D109N and T190M. Small Angle X-ray Scattering analysis reveals that these mutations affect the structures in solution, altering the distribution between compact and more elongated conformations. These results, supported by biochemical and biophysical assays, point to an altered structural flexibility of the mutant α-DG N-terminal region that may have repercussions on its interaction with LARGE and/or other DG-modifying enzymes, eventually reducing their catalytic efficiency.

## Introduction

Dystroglycan (DG), a ubiquitous membrane receptor belonging to the glycoprotein complex associated to dystrophin, plays a crucial role in the stability of the plasma membrane, especially in skeletal muscle tissues where it is highly expressed [1]. A single gene, *DAG1*, encodes for a protein precursor, which is proteolytically cleaved in two subunits, alpha-dystroglycan (α-DG), located at the extracellular side of the plasma membrane where it binds several extracellular matrix proteins, and beta-dystroglycan (β-DG), which is a transmembrane protein that interacts with dystrophin in the cytoplasm [2]. Many studies have been devoted to the structural characterization of the α-DG subunit. A pioneering electron microscopy study of α-DG revealed its dumbbell-like shape [3]. The molecular structure of the N-terminal region of α-DG (α-DG-Nt) was later determined at high resolution by X-ray crystallography, revealing the presence of two domains, an immunoglobulin-like (Ig-like) domain and a domain similar to the small subunit ribosomal protein S6 of *T*. *termophilus* (S6 domain), connected by a flexible loop [4]. The high resolution structure of the α-DG C-terminal domain is still elusive, although a computational study envisaged that this domain is also likely to possess an Ig-like fold, followed by a disordered part at the C-terminus [5]. A biochemical characterization confirmed a highly disordered conformation for the mucin-like central domain of α-DG [6], that is decorated and stabilized *in vivo* by specific O-mannosyl glycans built by a complex array of enzymes [7]. Among these, particularly worth of mention is the bifunctional Like-acetylglucosaminyltransferase (LARGE), which adds the repeating heterodisaccharide [-glucuronic acid-β1,3-xylose-α1,3-]_n_ to a glycan anchored at Thr317 and Thr319 in the mucin-like domain [8]. This region of the protein plays a crucial functional role, as its protruding carbohydrate moieties are known to mediate the interactions with laminin and other extracellular proteins containing laminin globular (LG) domains [9]. Indeed, many congenital muscular dystrophies, due to mutations affecting the genes encoding for glycosyltransferases belonging to the glycosylation pathway of α-DG, are characterized by hypoglycosylated forms of α-DG that are unable to interact with laminin, compromising the stability of sarcolemma [10]. Noteworthy, it has been shown that α-DG-Nt is necessary for recruiting LARGE [11] and some pathological conditions that are caused by missense mutations in the DAG1 map within the α-DG-Nt.

The first identified mutation (T192M), associated to a form of limb-girdle muscular dystrophy and cognitive impairment, prevents the correct glycosylation of α-DG resulting in impaired laminin binding. It was shown that the T192M mutation weakens the interaction between α-DG-Nt and LARGE, strongly reducing the ability of the latter to decorate α-DG with the proper carbohydrate moiety [12]. More recently, two additional mutations, namely V74I and D111N, located on the Ig-like domain surface facing the S6 domain within α-DG-Nt, have been found in a seven years old compound heterozygous patient, who displays mild muscular dystrophy and asymptomatic hyperCKemia. Also in this case, a biochemical analysis carried out on the muscular tissues of the patient revealed the presence of a hypoglycosylated form of α-DG that cannot bind to laminin [13].

In order to investigate the molecular mechanisms underlining these diseases, we analyzed the impact of the three afore mentioned pathological missense mutations on the conformational stability and on the overall structure of α-DG-Nt. In particular, we focused on the murine α-DG-Nt region, fully validated as a model for the human counterpart [14], carrying the three pathological missense mutations V72I, D109N and T190M that correspond to their topological counterparts V74I, D111N and T192M in human DG.

Even though the missense mutations V72I and D109N do not alter the overall fold of α-DG-Nt, as previously assessed by X-ray crystallography for the mutant T190M [15], Small Angle X-ray Scattering (SAXS) analysis (supported by limited proteolysis and Differential Scanning Fluorimetry (DSF) experiments) highlights a more complex dynamic behavior of α-DG-Nt in solution that is remarkably affected by the three pathological mutations. The resulting altered conformations may impact on the interactions of α-DG with LARGE, negatively influencing LARGE recruiting and ultimately the proper maturation of α-DG.

## Materials and methods

### DNA constructs, site-directed mutagenesis and protein expression and purification

The point mutations V72I and D109N were introduced within the murine α-DG(50–313) construct, carrying the additional mutation R166H, to improve its proteolytical stability, (hereinafter WT), cloned in pHis-Trx [4], by the QuikChange site-directed mutagenesis kit (Stratagene) as previously reported for T190M [15]. The following primers were employed to insert the two mutations:

V72I Forward: 5’-CCTGATGGCACGGCTGTCATCGGGCGCTCATTTCGAGTG-3’
V72I Reverse: 5’-CACTCGAAATGAGCGCCCGATGACAGCCGTGCCATCAGG-3’
D109N Forward: 5’-CCATCTTGGCTGCACTGGAACTCACAGAGCCACACCCTG-3’
D109N Reverse: 5’-CAGGGTGTGGCTCTGTGAGTTCCAGTGCAGCCAAGATGG-3’.

All constructs were verified by automated sequencing.

WT and the three pathological mutant proteins were expressed as N-terminal His_6_-tagged thioredoxin fusion products, containing a thrombin cleavage site, and purified before and after thrombin cleavage according to the previously described protocol [15].

### Differential scanning fluorimetry (DSF)

DSF experiments were performed using an excitation wavelength of 470–505 nm and an emission wavelength of 540–700 nm in a CFX96 Touch Biorad real-time PCR instrument (Bio-Rad) with a temperature gradient from 20 to 90°C in 0.2°C/ min increments. The final protein concentration was 0.5 mg/ mL in 20 mM Tris, 150 mM NaCl pH 7.5 (in the case of T190M the buffer was supplemented with 2.5% glycerol) and 90 × SYPRO Orange (Sigma). Experiments were carried out in triplicate and the averaged curves have been analyzed: melting temperatures (T_m_) were calculated by fitting the sigmoidal melt curves to the Boltzmann equation (S1 Fig) [16].

### Limited proteolysis

WT and its pathological mutants V72I and D109N, at a final concentration of 0.85 mg/ mL, were subjected to limited proteolysis with either α-chymotrypsin and trypsin at a final concentration of 2 μg/ mL. Proteolytic digestions were performed at 37°C and were stopped after 1, 5, 10, 20, 40 and 60 min by adding SDS sample buffer to aliquots of the reaction mixtures. The samples were analyzed by 15% SDS-PAGE [17] and Coomassie staining.

### Crystallization, data collection, structure solution and refinement

Crystals of D109N were grown by using the hanging-drop vapour diffusion method, exploring conditions similar to those used for both WT and T190M [4,15]. Drops were prepared by mixing 1 μL of protein solution (5.5 mg/ mL in 25 mM Tris, 150 mM NaCl, pH 7.5) with 1 μL of precipitant solution (0.6–1.4 M citrate buffer; pH 6.8–7.2) and equilibrated against the reservoirs containing 0.7 mL of the precipitant solution. Fully-grown crystals were obtained after two weeks at 0.7 M citrate buffer and pH 7.0, at the temperature of 277 K. Crystals of V72I did not grow under the same conditions of D109N and were obtained by using the cross-streak-seeding method. D109N crystals were used as seeds source and crystallization conditions between 0.6–1.4 M citrate buffer and pH between 6.8–7.2 were explored. Drops were prepared by mixing 1 μL of protein solution (5.0 mg/ mL in 25 mM Tris, 150 mM NaCl and pH 7.5) with 1 μL of precipitant solution; the drops were equilibrated against 0.7 mL of the precipitant solution at 277 K for 3–6 days, before seeding. Fully-grown crystals were obtained after 10–15 days after seeding (best crystals obtained at 0.8 M citrate buffer, pH 7.2). Repeated streak-seeding (2–3 times) at optimal precipitant conditions improved the crystal quality.

Data collections were carried out at the XRD1 beamline at ELETTRA (Trieste, Italy) [18,19] using Pilatus 2M (Dectris) detector and 1.00 Å (D109N) and 0.976 (V72I) wavelength. Data collection were carried out at 100 K. Crystals were quickly dipped into a cryoprotectant solution (25% v/v ethylene glycol added to the precipitant solution) and then frozen directly under a 100 K nitrogen gas stream.

Indexing, integration and data reduction of the diffraction data were carried out by using the XDS program [20]. Two data-set of highly isomorphous V72I crystals were merged with XSCALE [20] for higher data completeness. Data reduction statistics of D109N and V72I datasets are reported in Table 1.

**Table 1:** X-ray diffraction: Data collection and model refinement statistics.

	D109N	V72I
Space group	H3	H3
Unit-cell parameters (Å)		
***a***	71.75	71.81
***c***	144.00	143.99
Molecules per asymmetric unit	1	1
Wavelength (Å)	1.0000	0.9763
Resolution (Å)	48.0–1.70	35.9–1.80
Highest resolution shell (Å)^a^	1.76–1.70	1.86–1.80
Total observations	237820	294745
Unique reflections	30354	25641
R_merge_ (%)^b^	0.096 (1.098) ^a^	0.105 (1.301) ^a^
CC_1/2_^c^	0.997 (0.883) ^a^	0.999 (0.862) ^a^
<I/ σ (I)>	12.0 (1.9) ^a^	15.8 (2.1) ^a^
Completeness (%)	99.7 (97.9) ^a^	99.9 (99.4) ^a^
Redundancy	7.8 (7.5) ^a^	11.5 (11.3) ^a^
**Refinement**		
Number of reflections (work-set)	30326 (3042) ^a^	25623 (2540) ^a^
Number of reflections (test-set)	1516 (152) ^a^	1282 (127) ^a^
R_work_^d^	0.159 (0.349) ^a^	0.154 (0.292) ^a^
R_free_^e^	0.183 (0.360) ^a^	0.182 (0.315) ^a^
Number of non-H atoms		
Protein	1765	1738
Waters	196	172
Organic	15	19
Average isotropic B factors (Å^2^)		
Protein	33.5	36.2
Solvent	39.9	42.8
R.m.s. deviation		
Bond length (Å)	0.013	0.016
Angle (deg)	1.19	1.34
Ramachandran plot		
favored regions (%)	98	98
allowed regions (%)	2	2
disallowed regions (%)	0	0

^a^ Values in parenthesis are given for the highest resolution shell

^b^ R_merge_ = ∑_hkl_∑_j_│I_hkl, j_-<I_hkl_>│/ ∑_hkl_∑_j_ I_hkl_, _j_

^c^ CC_1/2_ values for *I*_mean_ are calculated by splitting the data randomly in half

^d^ R_work_ = ∑_work-set_│F_obs_-F_cal_│/ ∑_work-set_ F_obs_

^e^ R_free_ = ∑_test-set_│F_obs_-F_cal_│/ ∑_test-set_ F_obs_; Test-set: Randomly selected 5% of reflections excluded from the structure refinement.

The structure solution of both D109N and V72I were obtained by Molecular Replacement, using the WT crystal structure (PDB ID: 1U2C [4]) as search-model and PHASER [21] computer program as implemented in PHENIX [22] crystallographic package. Rigid-body refinement was initially carried out, followed by a simulated-annealing step. Several cycles of crystallographic refinement, including positional refinement, Translation-Libration-Screw (TLS) and individual B-factors refinement were alternated with the manual rebuilding of the structure by using the COOT software [23]. All the refinement cycles were carried out by using *phenix*.*refine* [24]. Solvent molecules were added to the model by using the automatic search protocol available in *phenix*.*refine* and manually checked before being included in the final model. Protein stereochemistry was monitored throughout the refinement process and during manual rebuilding with MolProbity [25]. Statistics of the crystallographic refinement are reported in Table 1. Various CCP4 [26] utility programs were used throughout the crystallographic study. Molecular diagrams were prepared using the PyMol Molecular Graphics System [27]. The PDB codes of the deposited structures are 5N30 (V72I) and 5N4H (D109N).

### Small-angle X-ray scattering measures and data processing

As previously reported for the WT protein [14], SAXS experiments for T190M were carried out at the BM29 beamline [28] of the European Synchrotron Radiation Facility (Grenoble, France) as 10 x 1 s exposures using a Pilatus 1M (Dectris) detector, with a sample-detector distance of 2.87 m and a wavelength of 0.99 Å. SAXS data for V72I and D109N were collected on the P12 beamline EMBL SAXS-WAXS at PETRAIII/DESY [29] (Hamburg, Germany) as 20 x 0.05 s exposures using a Pilatus 2M (Dectris) detector, sample-detector distance 3.00 m, wavelength 1.24 Å. Measurements were performed at six different concentrations (the ranges are reported in S2 Table) in 20 mM Tris, 150 mM NaCl pH 7.5 (supplemented with 2.5% glycerol in the case of T190M); the protein concentration was calculated using molar extinction coefficient at 280 nm (26595 M^-1^ cm^-1^) and measuring each sample dilution by a nanodrop spectrophotometer. No radiation damage effects were detected comparing the scattering curves of the collected frames.

Data were merged for each sample after normalization to the intensity of the transmitted beam. Subtraction of the scattering of the buffer and the following processing steps were carried out with PRIMUS [30] from the ATSAS 2.6.0 program package [31].

The radius of gyration R_g_ of the solute proteins and the forward scattering I(0) were evaluated by the Guinier approximation (1939) at very small angles (s < 1.3/Rg), assuming that the intensity is represented as I(s) = I(0)* exp(-1/3(R_g_*s)^2^) (S2 Fig), and from the entire scattering pattern by the program GNOM [32], that was used to compute also pair distance distribution functions of the particles p(r) and the maximum sizes D_max_. Molecular weights (MM) were evaluated by comparison of the calculated I(0) values with that of the standard solution of bovine serum albumin (MM 66 kDa). The excluded volume of the hydrated protein molecule (V_p_) was obtained using the Porod approximation [33]: $V_{p}=\frac{2\pi^{2}I(0)}{\int I_{exp}(s)s^{2}ds}$.

Low resolution shapes of the three mutants were produced by the *ab initio* program DAMMIN [34] that employs a simulated annealing procedure to build a compact dummy atoms (beads) model that fits the experimental data I_exp_(s) to minimize the discrepancy: $\chi^{2}=\frac{1}{N-1}\sum_{j}{\left[\frac{I_{exp}(s_{j})-cI_{calc}(s_{j})}{\sigma (s_{j})}\right]}^{2}$.

Ten independent DAMMIN runs were performed for each data set in the “slow” mode with no symmetry assumptions (P1 symmetry): the resulting models were superimposed using the program SUPCOMB [35] and averaged using DAMAVER [36] to identify the most typical models representing the global shape of the three mutants in solution. The normalized spatial discrepancy parameter (NSD) [35] obtained from DAMAVER indicated the similarity between models used for average calculations. NSD values ≤ 1.0 are expected for similar models.

Rigid-body modeling was performed using CORAL [31], where the high-resolution X-ray crystal structures of the Ig-like and S6 domains of the three mutants were used as inputs in the refinement calculations. This program was employed to refine the spatial arrangement of the two domains for the three mutants and to model clash-free configurations of the missing portions of polypeptide chains (around 10 aminoacids at both N-terminal and C-terminal and the missing linker between the Ig-like and S6 domains). CRYSOL [37] was used to evaluate the fits to the experimental data of the respective X-ray crystal structures; CORAL was also used to generate the approximate conformations of the missing regions keeping fixed the two domains as in the respective X-ray crystal structures.

Inter-domain flexibility and size distribution of possible conformers for the three mutants were quantitatively assessed by the ensemble optimization method (EOM) [38]. This method assumes the existence of a mixture of conformations in solution; the average scattering of the mixture fits the experimental data. In EOM, an initial random pool of 10000 conformers was generated. In these conformers the modeled linker residues and the modeled N-terminal and C-terminal stretches were allowed to have random-coil conformations and the S6 domain and the Ig-like domains, obtained by the respective crystal structures of the three mutants, were used as rigid bodies. The theoretical scattering was calculated for each generated model by CRYSOL. A genetic algorithm (GAJOE) was used to select an ensemble of conformations whose mixture best fitted the experimental data. Multiple runs of EOM were performed and the obtained subsets were analyzed to yield the R_g_ distributions in the selected ensembles. Once each ensemble is determined, the corresponding Shannon Entropy, reported as R_flex_, provides a quantitative measure of flexibility [39]. Using R_flex_, each ensemble distribution can be numerically compared to that of the respective random pool, the latter representing a reference for flexibility. The complementary metric R_σ_ (i.e. the ratio of the standard deviation between the ensemble and the pool distributions) allows to identify potential spurious solutions: values close to 1.0 are obtained when the ensemble distribution largely reproduces the conformational space of the random pool.

### Sequence alignment

Multiple sequence alignments of protein sequences were constructed in Clustal omega via the resources of EMBL/EBI (http://www.ebi.ac.uk/Tools/msa/clustalo/).

## Results and discussion

### V72I and D109N mutations determine small local variations into the crystal structure of N-terminal region of α-DG

**Fig 1:**
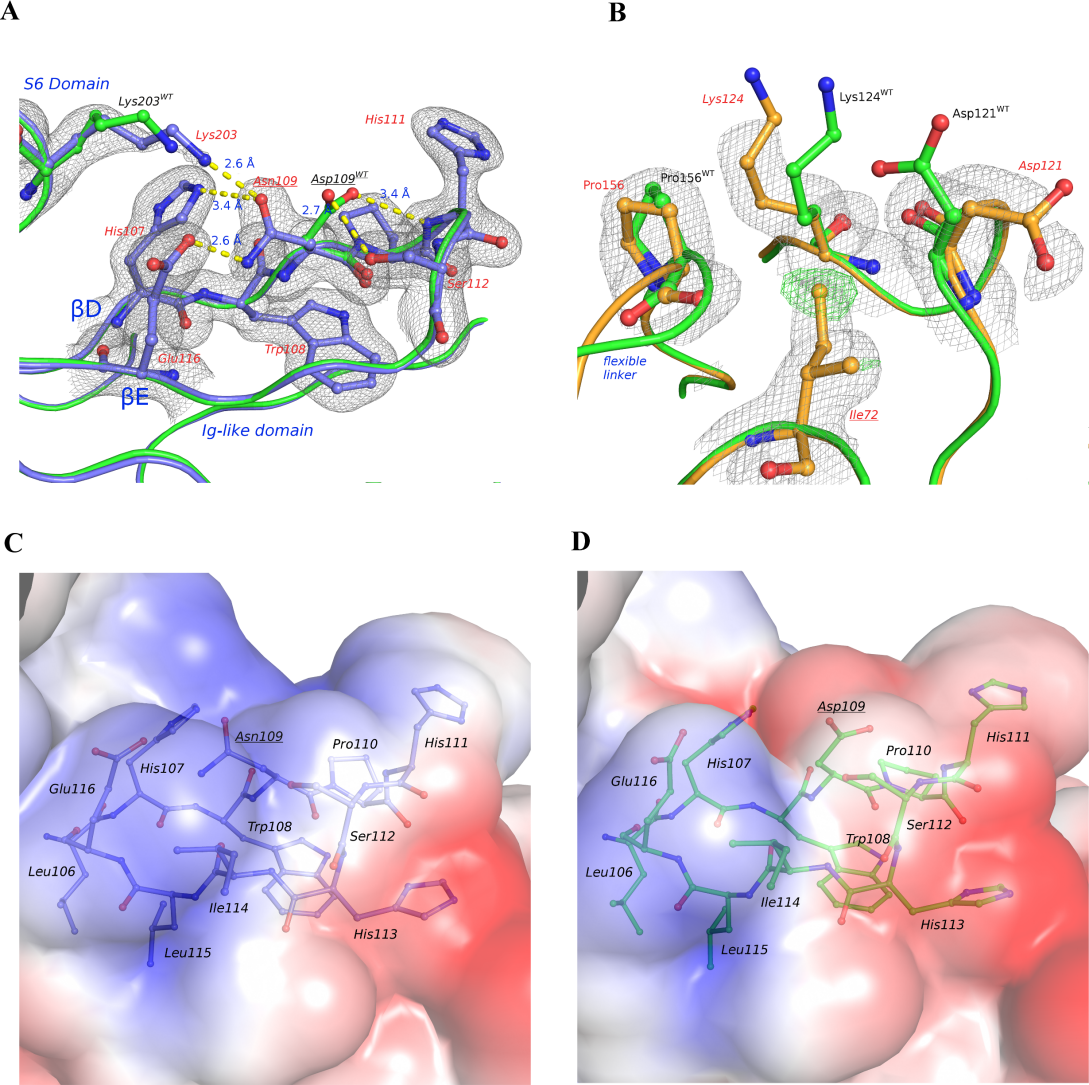
V72I and D109N crystal structures. Details of the superimposition of the WT with D109N (**A**) and V72I crystal structures (**B**). WT, D109N and V72I are represented as ribbon. Selected residues are represented as stick-and-ball, with WT (PDB ID: 1U2C) colored in green, D109N colored in blue and V72I colored in orange. Residues belonging to WT are labelled with WT superscript in panels (**A)** and (**B)**. The models are overlaid by D109N σ_A_-weighted 2F_o_-F_c_ map (contoured at 1.0 σ and colored in grey) (**A**) and by V72I σ_A_-weighted 2F_o_-F_c_ map (contoured at 1.0 σ and colored in grey) and F_o_-F_c_ map (contoured at 3.0 σ and colored in green) (**B**). Electrostatic potential maps of WT and D109N are represented in panel **(C)** and (**D**) respectively. The electrostatic potential (in *k*_*b*_T/*e*_*c*_ units) is mapped on solvent-accessible surface of the WT and D109N pathological mutant accessible surfaces. Negative potential is colored in blue, positive potential in red. Color scale varies between -2 and +2. Molecular models (stretch 105–116) are represented as ribbons with selected residues depicted as stick-and-ball.

The crystal structures of D109N and V72I have been determined to a resolution of 1.70 Å and 1.80 Å, respectively. Similarly to what observed for T190M [15], residues 50–58 and 305–315, as well as residues 163–179 (V72I) or 164–179 (D109N), were missing in the D109N and V72I final models. Residues 89–91 and 181–185 belong to highly mobile loops thus showing an inherent lower quality electron density. In addition, the region encompassing residues 159–162 displays evidences of less populated conformations in both structures, which could not be confidently modeled during the structure refinement. That said, both D109N and V72I structures show the typical overall folding of α-DG-Nt [4]. The Ig-like and S6 domain are linked by a flexible loop encompassing residues 159–179, which is only partially visible in the present models. The two pathological point mutations introduced in α-DG-Nt were identified according to the 2F_o_-F_c_ and F_o_-F_c_ maps (Fig 1A and 1B) and by their effects on the local molecular geometry.

According to the crystal structures, the two point mutations do not induce significant changes in the domains fold nor in their mutual orientation when compared with WT. Indeed, the superposition of the structure of WT with those of D109N and V72I gives root mean square deviations of 0.353 Å (calculated on 227 Cα) and 0.417 Å (calculated on 226 Cα), respectively, in line with the superposition of the WT and T190M structures (0.758 Å) [15].

The Asp109→Asn mutation introduces local changes on the molecular geometry that are quite evident in the final refined model. The Asn109 side chain rotates nearly 120° around Cα-Cβ with respect to the orientation held by Asp109 in the WT. The conformation assumed by Asn109 allows for the establishment of H-bonds with Lys203, Glu116 and His107. Accordingly, Asn109 is not engaged anymore in H-bonds with Ser112 and His111 (Fig 1A) as Asp109 is in WT. Quite surprisingly, this newly established H-bonds network does not affect the geometry of the now untied turn including His111 and Ser112 that connect the two β-strands D and E.

Furthermore, Asn109 also interacts with Lys203, which belongs to the S6 domain. According to its electron density and B-factors, Lys203 is inherently rather flexible and it is plausible that the strength of the interaction between Lys203 and Asn109 would be rather weak. In addition, the H-bond network involving Asn109, Glu116 and Lys203 in D109N abolishes the interaction between Lys203 and Glu116 observed in the WT structure. Hence, it is likely that the mutated H-bond network in D109N alters the tightness of the interaction between the Ig-like and the S6 domains. In this respect, it must be emphasized that the Asp109→Asn mutation not only changes the H-bond network involving residue 109, but also modifies the local electrostatic potential (Fig 1C and 1D), so that the interaction of Asn109 with the surrounding positively charged residues like Lys203 and Arg77 is affected. This is confirmed by the presence of two water molecules (HOH 548 and 621) absent in WT structure and coordinating Arg77, thus filling the void left by the Asn109 side-chain relocation. It is therefore likely that the change in electrostatic potential observed in D109N also affects the tightness of the interaction between the Ig-like and the S6 domains.

According to the refined crystallographic model, the pathological mutation Val72→Ile has no effect on the overall conformation of α-DG-Nt, but in this case also the local effects on the structure around the mutated residue are quite limited. The bulkier isoleucine residue might perturb the N-terminal part of the flexible linker connecting the two domains, with some effects on Lys124 and Asp121 (Fig 1B and S3 Fig). These residues are solvent exposed and display, in WT, a remarkable mobility. It is worth noting that the linker region is one of the structural elements of α-DG-Nt with the highest flexibility: it is therefore difficult to confidently assess the effect of the Val72→Ile mutation on the linker conformation, considering that it is only partially defined in the refined V72I model. It is also worth of note that the conformation of the modeled part of the linker in V72I is essentially the same as that observed in T190M, but rather different from what observed in WT, pointing to an effect not related to the specific presence of Ile72.

### Conformational stability of the pathological mutants

Conformational stabilities of the three pathological mutants as compared to WT were assessed by limited proteolysis and DSF assays.

**Fig 2:**
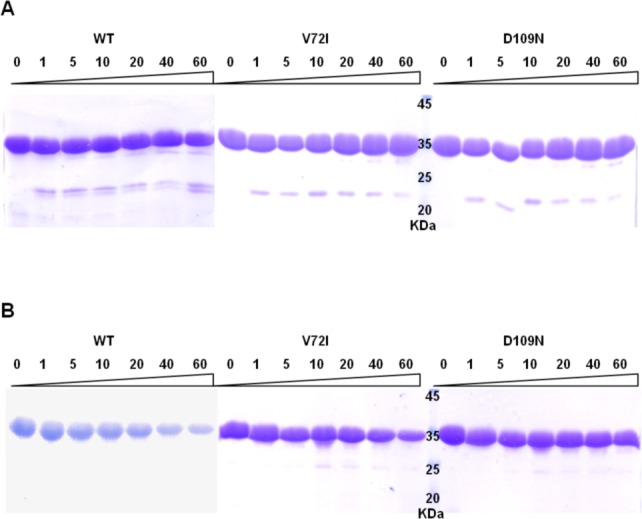
Limited proteolysis analysis. Protease digestion of α-DG-Nt WT and its pathological mutants V72I and D109N by α-chymotrypsin **(A**) and trypsin (**B**) for 1, 5, 10, 20, 40 and 60 min. Undigested samples served as the zero time point (0).

Considering that conformational parameters such as solvent accessibility and segmental mobility are known to correlate with exposed proteolytic sites [40], limited proteolysis analysis was performed with two different proteases (respectively α-chymotrypsin and trypsin) in order to identify potential flexible and exposed regions. The results shown in Fig 2 suggest only slight differences in susceptibility to proteolysis of V72I and D109N with respect to WT protein, not as striking as those recorded in a comparative tryptic analysis of the T190M mutant [15].

**Fig 3:**
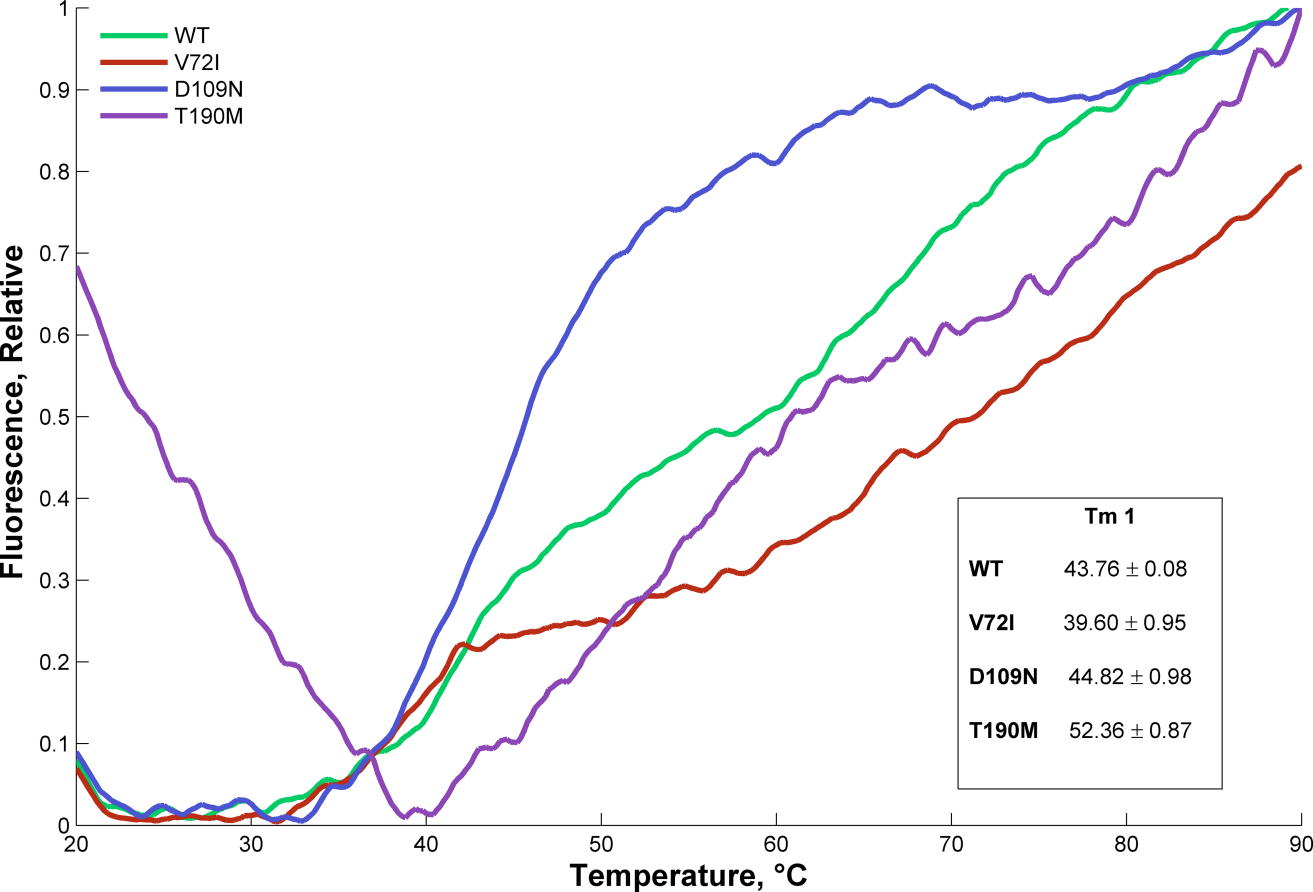
Thermal denaturation assay using DSF with SYPRO dye. Comparison of unfolding fluorescent curves for the WT α-DG-Nt and its pathological mutants; the melting temperature (T_m_) values from the Boltzmann Sigmoid fitting of the first transition of the curves are reported in the insert. Experiments were performed in triplicate and the averaged curves are shown.

In order to investigate further whether the pathological mutations are associated with variation in conformational stability, DSF was performed. This assay monitors thermally-induced protein unfolding by the binding of a fluorescent dye to the hydrophobic core of the protein as it becomes exposed, with an increase in fluorescence emission as the dye binds. From the unfolding curve thus the T_m_ of a protein can be calculated, which is indicative of its thermal and conformational stability: a shift towards lower temperatures in the T_m_ of a protein variant relative to that of the WT is evidence of its destabilization [16]. Therefore, the analysis of the DSF curves and T_m_s are especially useful for a qualitative comparison of the mutants thermal stabilities. Fig 3 compares the changes in fluorescent signal of the WT and mutant proteins during thermal unfolding in the presence of the dye. WT, V72I and D109N show very low and flat background fluorescence in the pre-transition region, while T190M is characterized by a quite high fluorescence in the pre-transition region, often symptom of exposed hydrophobic residues [41]. This finding is in accordance with the substitution of the polar side chain of Thr with a bulkier and apolar group such as the methylthio group of the Met that can create a binding site for the hydrophobic dye in the native state. Even if all the resulting denaturation curves point to a two-transitions unfolding process, as it has been observed in several proteins composed by two domains that independently fold, the profiles are dramatically different, suggesting that the single point mutations deeply influence the protein conformation/stability in solution. In particular, the first transition is much sharper (i.e. more cooperative) for V72I and D109N than for T190M and WT. The T_m_ values (summarized in the insert of Fig 3) could be calculated, by fitting the data to a Boltzmann Sigmoid only for the first transition since the second is not sufficiently defined. The fitting was especially problematic for the T190M case, due to its large pre-transition fluorescence. Two mutants (D109N and T190M) were characterized by an increase in T_m_ relative to WT, while for V72I a small decrease was observed. The second transition profiles suggest an increased T_m_ of the mutants with respect to the WT, although in this cases the T_m_ could not be calculated because it was not possible to reach the aggregation region (*i*.*e*. the endpoint of the transition), even increasing the final temperature to the upper range value of the instrument.

### Association state and overall size parameters of the pathological mutants in solution

**Fig 4:**
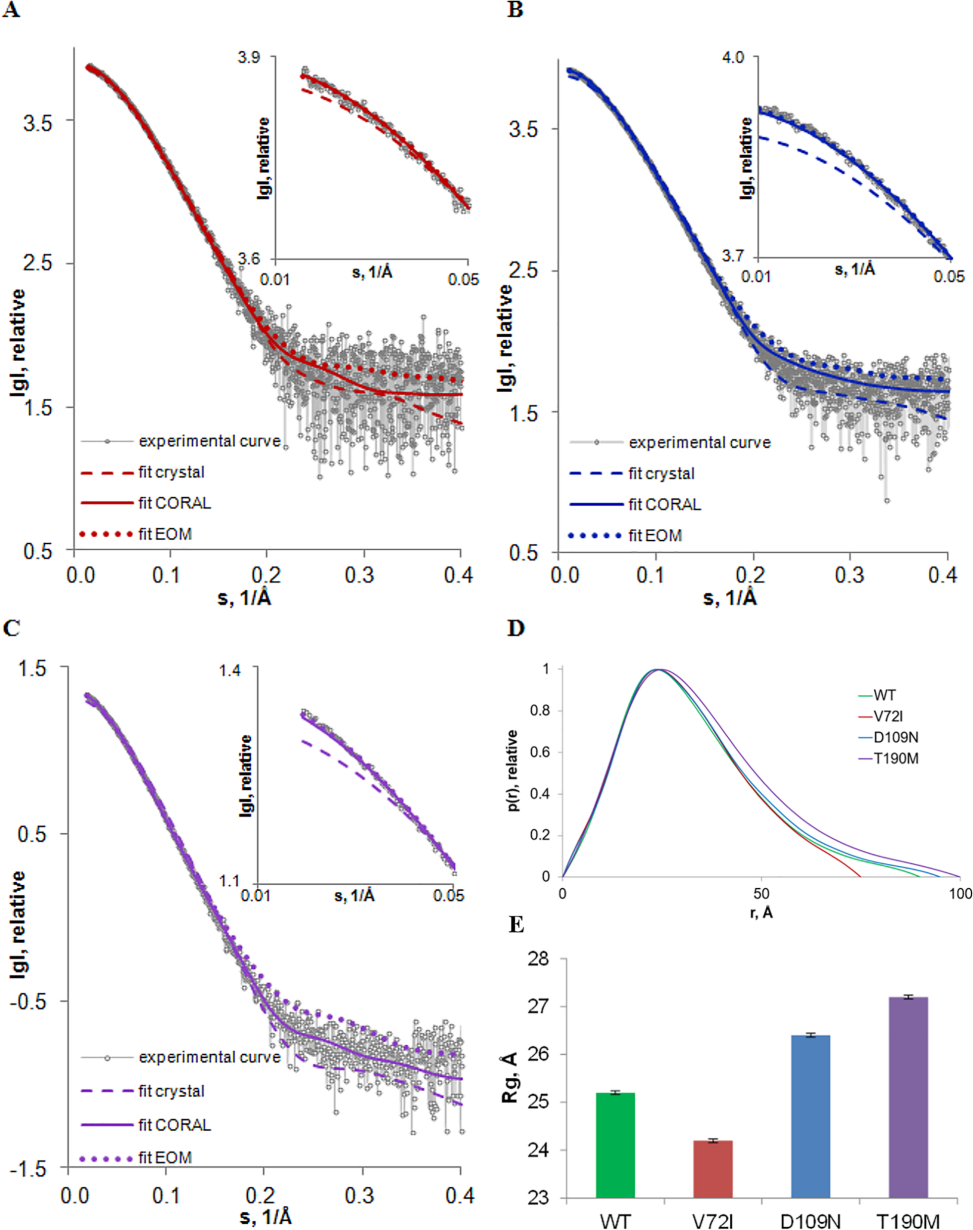
**Experimental X-ray scattering data and the obtained fits of the three pathological mutants of α-DG-Nt: A) V72I; B) D109N and C) T190M.** Experimental SAXS patterns, scattering calculated from the respective crystallographic models with added missing regions obtained by CORAL (fit crystal) keeping fixed the two domains and scattering calculated from models obtained by CORAL (fit CORAL) are plotted as indicated. The plots display the logarithm of the scattering intensity as a function of momentum transfer s = [4πsin(θ/2)]/λ (Å^-1^), where θ is the scattering angle and λ is the X-ray wavelength. The zoomed regions of these graphs at low angles are presented in the inserts. The comparison of the distance distribution functions obtained for the WT and the three pathological mutants of α-DG-Nt are presented in panel (**D**), the corresponding R_g_ values are plotted in panel (**E**).

In order to assess whether the single point mutations associated to pathological states impact on the conformation of α-DG-Nt in solution, SAXS experiments were performed on the three mutants at different concentrations (S1 Table) without observing systematic changes due to solute concentrations or any interparticle interaction (S4 Fig): therefore the analysis was performed on the curves collected for most concentrated samples. Fig 4 displays the processed scattering data collected for the highest concentration of each mutant, and S1 Table compares the resulting overall size parameters to previously collected WT data [14] (additional details on SAXS structural parameters are reported in S2 Table).

The V_p_ and the MM calculated for all mutants (S1 Table) were consistent with the values expected for a monomeric species even at relatively high (up to 7 mg/ml) concentrations and are in agreement with the MM estimated from the primary sequences (around 28.5 kDa).

The computed distance distribution functions p(r) are compared to the profile obtained for the WT protein in Fig 4D. All the profiles display a single peak with a tail, a pattern indicative of proteins having elongated structures, but the significant change in the maximum dimensions of the mutants, combined with a significant variation in the radius of gyration (R_g_) (Fig 4E and S1 Table), are further evidence that the pathological point mutations affect the conformation of α-DG-Nt.

In details, the mutations Asp109→Asn and Thr190→Met led to a significant increase in the maximum dimensions (with D_max_ increased from 90 ± 3Å for WT to 95 ± 3Å and to 100 ± 3Å for D109N and T190M respectively) and in the R_g_ values (from 25.20 ± 0.04Å for the WT to 26.40 ± 0.04Å and to 27.20 ± 0.04Å for D109N and T190M, respectively), suggesting a more extended shape of the two pathological mutants compared to the WT protein. On the contrary, the single mutant V72I shows a decrease both in D_max_ (from 90 ± 3Å for WT to 80 ± 2Å for V72I) and in the R_g_ value (from 25.20 ± 0.04Å for the WT to 24.20 ± 0.04Å for V72I), pointing to a more compact shape as compared to the WT protein.

Similarly to what determined by the SAXS experiments performed on the WT protein [14], all the mutants in solution have a significantly more elongated conformation than the respective crystallographic models (S5 Fig). Indeed, the scattering curves computed by the CRYSOL program [37] from the crystallographic models (PDB_IDs: 5N30 for V72I, 5N4H for D109N and 4WIK for T190M [15]) give a poor fit to the experimental data (not shown), even after the reconstruction of the missing regions (around 10 aminoacids at both N-terminal and C-terminal and the missing linker between the two domains that are kept fixed) using the program CORAL [31] (S1 Table Χ_crystal_, Fig 4 fit_crystal_ with the zoomed portions at low angles in the inserts). Despite SAXS cannot characterize a molecular structure at a resolution level comparable to that provided by X-ray crystallography, it allows to define structural models in solution devoid of the constrains imposed by the packing forces featuring crystal structures. In our models packing forces may influence the relative orientation of the Ig-like and S6 domains that in solution gain a certain degree of freedom assured by the highly flexible loop connecting them. This may have relevant effects on the overall conformation of the α-DG N-terminal domain that is expected to influence the shape of the molecule and, accordingly, the very low-resolution part of the scattering curve.

### Effects of the pathological mutations on the shape of the N-terminal region of α-DG in solution

The macromolecular shapes of the three mutant proteins in solution have been reconstructed in parallel by *ab initio* modeling and by rigid-body modeling.

**Fig 5:**
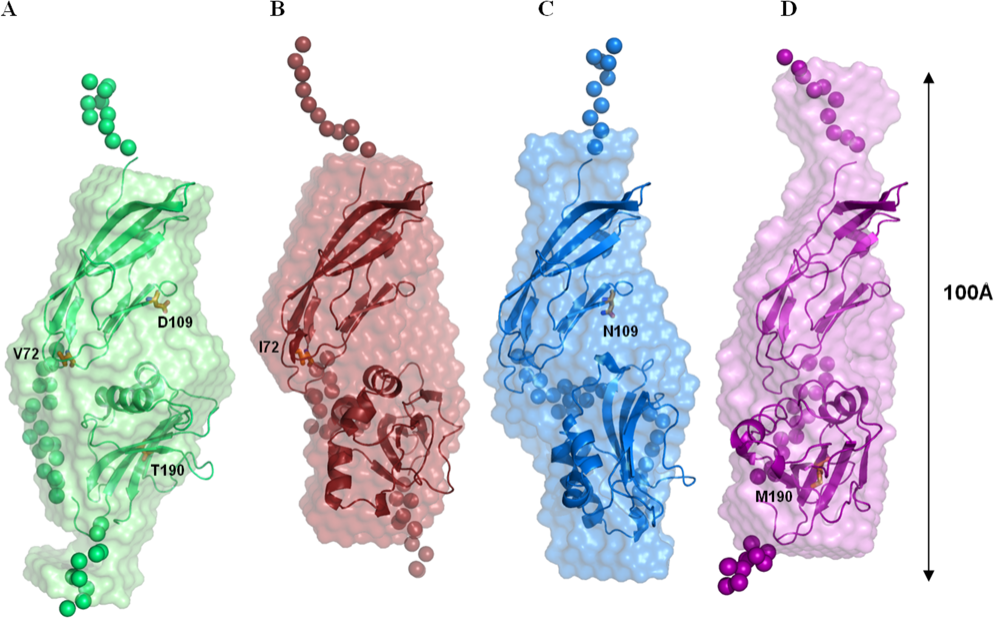
Comparison of the structural models of the WT and the three pathological mutants of α-DG-Nt. Averaged and filtered *ab initio* bead models obtained with DAMMIN (semitransparent surfaces) overlaid to the typical CORAL models (green, red, blue and violet cartoon representations for the folded Ig-like and S6 domains, spheres for the restored missing fragments of WT (A), V72I (B), D109N (C) and T190M (D), respectively). Residues involved in the three pathological mutations are represented by sticks and colored in orange.

All the 10 independent solutions for each mutant (Fig 5), reconstructed from the X-ray scattering data using DAMMIN [34], showed a good fit to the experimental curves (Χ_ab-initio_ in S1 Table). Thus they were averaged to obtain the final low-resolution models of the three mutant proteins with quite low NSD values (0.698 ± 0.018, 0.570 ± 0.039 and 0.533 ± 0.031, for V72I, D109N and T190M, respectively), indicating that the multiple solutions built by the program are very similar to each other. The comparison of the resulting *ab initio* models with that of the WT suggests that small but significant rearrangements (at low resolution) in the orientation of the two domains occur, leading to a more extended structure for D109N and T190M and to a more compact conformation for V72I (Fig 5).

In order to get more detailed information exploiting the respective high-resolution crystal structures, with the Ig-like and S6 domains treated as rigid bodies, the program CORAL [31] was used to optimize the relative orientations of the two domains and to reconstruct the missing regions. Multiple runs were performed and yielded variable conformations, all providing good fits to the experimental data (i.e. Χ in the range of 1.0–1.1 for V72I, 1.1–1.6 for D109N and 1.1–1.7 for T190M). Even if this variety of configurations suggests a significant flexibility of this region (see below for the discussion of the inter-domain flexibility), the good fit of the best rigid-body models of the mutants (Χ_CORAL_ in S1 Table and fit_CORAL_ in Fig 4) indicates that these models provide a good representation of the average conformations. These results confirm that the pathological mutant proteins have a more extended shape in solution than that observed in the crystal lattice (as previously assessed for the WT protein). One of the possible reasons for that is the absence of the packing forces in solution together with the two-domain structure of the proteins which are connected by the flexible linker. In this respect it is interesting to note that the positions of the centers of masses of the S6 domains in all the crystallographic models nicely overlap (S6 Fig) and are much closer to the center of mass of the Ig-like domain than the respective CORAL models. As a quantitative measure of structure compactness, the distances between centers of masses of the two domains in the crystallographic models (29.9 Å, 29.7 Å, 29.6 Å and 29.6 Å in the WT, D109N, V72I and T190M models, respectively) have been compared to the respective CORAL models (34.4 Å, 36.6 Å, 33.0 Å and 37.4 Å in the WT, D109N, V72I and T190M models, respectively), confirming the existence in solution of rather elongated conformations that are more flexible than what their crystal structures suggested. Moreover, this comparison highlights that the three mutations significantly impact on the protein conformation, in agreement with what observed for the respective SAXS model envelopes.

It is interesting to note that, comparing the CORAL models of the three mutants to that of the WT one, the S6 domain appears to be rotated to different extents around the Ig-like domain. In the case of both V72I and D109N, the mutated residues (whose lateral chains are highlighted by orange sticks in Fig 5) are mapped on the surface of the Ig-like domain facing the S6 domain, and might induce a rearrangement in the mutual orientations of the two domains either directly or by an indirect effect mediated by the linker. Such an explanation cannot be invoked for the T190M mutation, which is located on the external surface of the S6 domain.

### Pathological mutations alter the flexibility of the N-terminal region of α-DG in solution

The presence of disordered regions in the crystal structure of the WT protein, has been reported to indicate a certain degree of inter-domain flexibility that could account for the observed conformational variability in solution [14].

**Fig 6:**
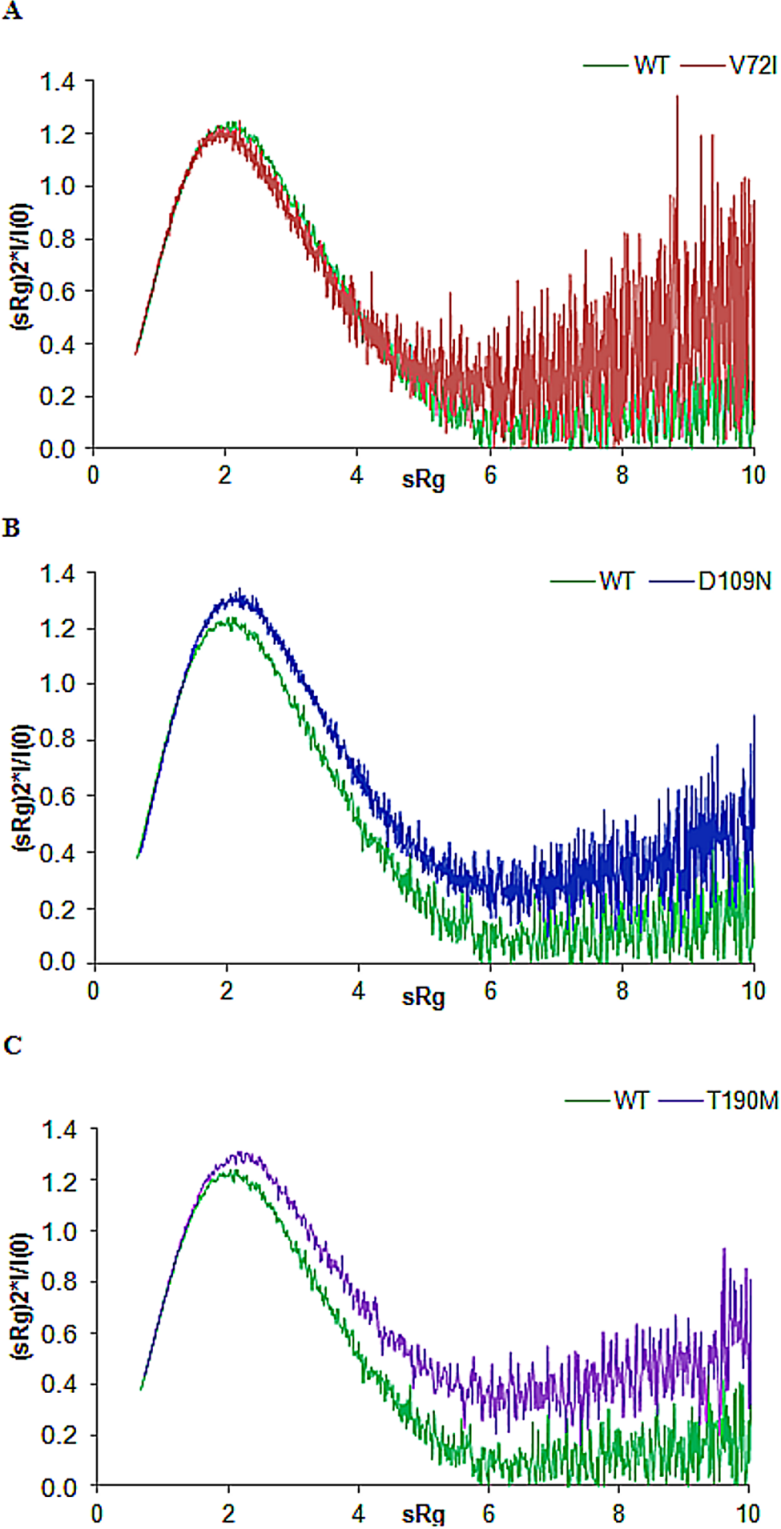
Normalized Kratky plots. Comparison of Kratky plots for the pathological mutants A) V72I, B) D109N and C) T190M with that of WT α-DG-Nt.

The occurrence of possibly increased inter-domain flexibility in the three pathological mutants with respect to the WT was investigated using two different approaches. An essentially qualitative approach, the so called normalized Kratky plot [42], allows to directly compare objects of different shape and size. In such a plot, folded compact globular proteins provide a bell-shaped curve at low angles with a maximum at s*R_g_ 1.75 [42]. Deviations from this behavior point to particle flexibility as in the case of the WT protein, whose maximum falls at s*R_g_ 2.1 (Fig 6). The plots for D109N and T190M both show a broadening of the bell-shaped curve and a shift of the maxima to larger s*R_g_ values, expected for more extended and flexible particles, while for V72I the curve is slightly sharper. In any case, the plots of the three mutant proteins are characterized by upward trends at higher s*R_g_ values (*i*.*e*. higher scattering angles) as compared to the more downward trend observed for the WT, an indication of increased flexibility in these former.

In the second, more quantitative approach, the ensemble optimization method (EOM) [38] was employed in order to analyze inter-domain flexibility and size distribution of possible multiple configurations in solution and to obtain typical optimized ensembles with a good fitting to the experimental scattering data (fit_EOM_ in Fig 4 and Χ_EOM_ S1 Table).

**Fig 7:**
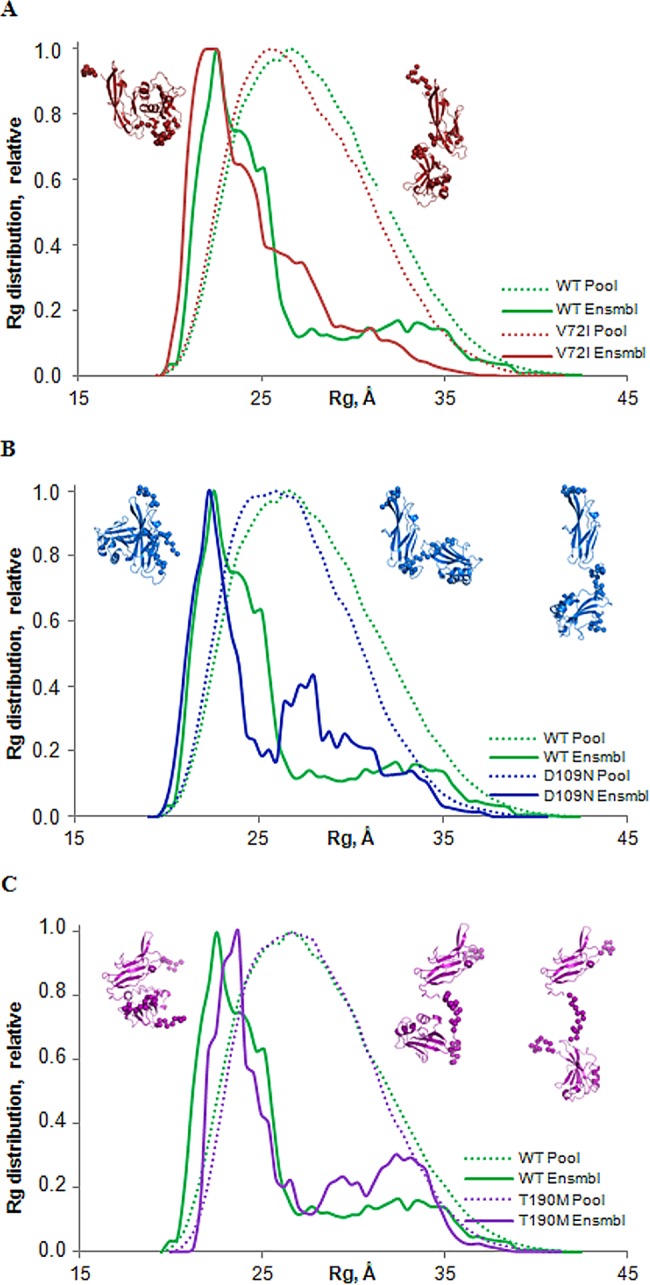
R_g_ distributions for the EOM models. Comparison of R_g_ distributions of the pathological mutants A) V72I, B) D109N and C) T190M with that of WT α-DG-Nt. The distributions for the initial random pools of models are shown by dot lines and the solid lines correspond to the selected ensembles. The representative conformations are shown near the distributions: compact on the left, intermediate in the middle and extended conformation on the right. Folded domains are depicted as cartoons (V72I in red, to D109N in blue and to T190M in violet); linkers and the N-terminal and C-terminal reconstructed regions are represented by spheres.

The EOM analysis for the three mutants are compared to WT in Fig 7 as a size distribution, plotting the R_g_ of the structures forming the initial random pool and the selected ensembles. The R_g_ distributions of these ensembles (Fig 7 solid lines) are nearly as broad as the distribution of randomly generated models (Fig 7 dashed lines) supporting the hypothesis of considerable inter-domain flexibility. Moreover the quantification of the flexibility of the WT protein (ensemble R_flex_ = 83.9% versus pool R_flex_ = 88.5%) and of the three mutants V72I (ensemble R_flex_ = 81.9% versus pool R_flex_ = 87.7%), D109N (ensemble R_flex_ = 85.8% versus pool R_flex_ = 86.6%) and T190M (ensemble R_flex_ = 82.7% versus pool R_flex_ = 88.8%) confirmed random motion of the Ig-like domain with respect to the S6 domain. The quality of the ensemble solutions were further validated by the values of R_σ_ that were close to 1.

However, it is interesting to note that the three mutants display alterations in the bimodal profile that characterizes the distribution of size observed for the WT protein (Fig 7 green solid lines). In detail for V72I the profile is mainly unimodal characterized by a broader single peak: even if its maximum is slightly shifted to more compact models with R_g_ about 21–25 Å (60.4% of the total population), there is an increased fraction of extended models with intermediate R_g_ around 25–30 Å. The distribution for D109N and T190M is still bimodal like the WT but beside the predominant fraction of relatively compact models, whose peak is sharper for D109N with R_g_ about 21–24 Å (43.1%), while slightly shifted to less compact models for T190M, with R_g_ about 21–27 Å (61.4%), both mutants show a significant increase in the population of elongated models with intermediate R_g_ around 25–30 Å. Indeed, although the small fraction of more extended models with R_g_ about 30–37 Å present in the WT (16.5%) can be still observed in D109N (15.3%) and is even increased in T190M (27.5%), but is scarcely represented in V72I (8.1%), all three mutants share a significant fraction with intermediate R_g_ around 25–30 Å (25.8%, 29.2% and 22.5% for V72I, D109N and T190M, respectively) that is remarkably less populated in the distribution of the WT protein (18.6%).

### Functional implications of the pathological mutations of α-DG

The present analysis of the conformational variability of the mutants of α-DG-Nt in solution at low resolution pinpoints to inter-domain flexibility as an important structural determinant mediating the pathological alterations induced by the missense point mutations, that in human result into primary dystroglycanopathies [12,13,43].

To this respect it is worth noting that in the presence of a single wild-type copy of the DG allele no haploinsufficiency, or in any case no phenotype involving DG has been reported in the parents and/ or family of the compound heterozygous patient studied by Dong and colleagues. This finding suggests there are no relevant dominant effects in this case [13] nor in the heterozygous relatives carrying the T192M mutation [12]. In accordance with the heterozygous compound human phenotype, apparently they have never been co-selected [44]. No single nucleotide polymorphisms have been reported in the human DG gene within the region corresponding to its N-terminal Ig-like domain [45,46]. However, it is interesting to note that the sequence alignments for representative members of vertebrate α-DG regions containing these three mutations (S3A Table for Val72 and Asp109 according to the murine Ig-like domain of α-DG and S3B Table for Thr 190) reveal that Val72 and Thr190 are highly conserved across evolution, at least in higher animal groups. On the contrary, Asp109 has been apparently subjected to a less stringent negative selection pressure. Indeed Reptiles and Amphibians show a conservative substitution (Asp→Glu in green in S3A Table) in position 109 corresponding to the murine α-DG; in Fish beside the same conservative substitutions, also not conservative mutations can be observed (in red in S3A Table); in Birds the same pathological mutation Asp→Asn seems to be quite common (in yellow in S3A Table). However, in chicken [47] no missense mutation hitting the α-DG-Nt has been described to cause dystroglycanopathies. Among Fish, in zebrafish, where DG knockdown has been reported to cause a muscular dystrophy [48], all these three positions are conserved, even if a missense mutation (V567D) has been reported only within the second C-terminal Ig-like domain of α-DG, completely abolishing its presence [49]. Most importantly these three positions are highly conserved in Mammals and in particular in dog and cat, where muscular dystrophies have been reported in which α-DG glycosylation [50] or expression levels [51] are respectively altered. Finally in humans these three α-DG pathological mutations are known to lead to hypoglycosylated α-DG and thus to primary dystroglycanopathies of different severity [12,13,43]. To bind its physiological ligands in ECM (laminin, agrin, perlecan) mature α-DG requires a correct glycosylation that depends on a complex pattern of post-translational modifications. Indeed, during maturation a cascade of enzymatic reactions leads to matriglycan [7], recently proposed name for the glycan section of α-DG that actually forms a bridge with the laminin LG domains. The impact of these pathological mutations on α-DG-Nt, which is known to assist the bifunctional glycosyltransferase LARGE during the matriglycan extension stage [7,11] might depend on their ability to negatively affect the interaction of α-DG with LARGE and/ or make α-DG-Nt unable to properly assist LARGE in its enzymatic activity. From a structural point of view the crystallographic structures here discussed point to local differences with respect to the WT structure. It cannot be excluded that the local alterations observed in D109N and V72I may negatively affect the molecular determinants of LARGE recognition and/ or binding by α-DG. Such a hypothesis clearly requires a more detailed structural study in order to be correctly addressed.

Despite showing similar crystal structures, the three pathological mutants here discussed display a strikingly different behavior in solution, not only from each other but also from the WT protein. It has already been observed that the crystallographic models do not fully account for α-DG-Nt structure in solution nor for its conformational variability [14]. The framework resulting from a previous SAXS study suggests that multiple conformations and their associated populations observed in solution are shared features of both human and mouse α-DG[14], pointing to functional implications for the α-DG structural plasticity.

Indeed, LARGE action requires different catalytic steps involving two distinct glycosyltransferases domains that in turn extend matriglycan [7,11,52]. LARGE activity leads to the synthesis of a glycan of remarkably high molecular weight, due to the decoration of the mucin-like region of α-DG with several [-glucuronic acid-β1,3-xylose-α1,3-] units, that in mature α-DG spans more than 100 nm in length [8]. It is safe to assume that α-DG-Nt would need a certain degree of conformational variability in order to assists LARGE into such a complex matriglycan elongation. The SAXS study here presented clearly indicate that the pathological point mutations studied, while not largely affecting the α-DG-Nt structure in crystal, do affect its conformation in solution. The effects on the average conformations of the three pathological α-DG mutants T192M, V72I and D109N clearly emerged from shape reconstruction and from rigid-body fitting. The impact of the pathological mutations on the α-DG-Nt conformation are further confirmed by the analysis of the conformational ensembles variability. EOM analysis reveals that the mutations do affect the bimodal partition observed in human and mouse α-DG-Nt, with a general increase in the mutants inter-domain flexibility.

It is worth noting that none of the mutations under analysis seem to affect significantly the overall stability of the DG core protein as far as its expression, trafficking and membrane targeting is concerned [12,13]. Interestingly, the increased conformational flexibility of the mutant proteins seems to be related to a gain in their thermal stability (with the exception of V72I), quantified by DSF. This result is quite unexpected as thermal stability of a protein is commonly associated to its structural rigidity. Nevertheless, an increasing number of studies arises the need to reconsider such a notion [53]. For example, comparing thermodynamic parameters of the unfolding process of thermostable and mesostable proteins, it has been found that thermostability may be attributed to reduced entropy changes between unfolded and folded states, due to an enhanced number of conformations that can be sampled by the folded protein, especially at higher temperatures. In other words, an increased conformational mobility of the folded state reduces the entropic contribution to the ΔG of the folded → unfolded transition, shifting melting temperature T_m_ towards higher values [54,55]. This may explain the increased T_m_ values measured for D109N and T190M, and the reduced T_m_ value of V72I, with respect to WT. Indeed, V72I populates conformational states that are more densely packed than those sampled by WT and its average conformation is more compact, as also supported by its lower maximum dimension (D_max_) and radius of gyration (R_g_) compared to WT. Moreover, the relative increased resistance to proteolysis, displayed by the mutant proteins, despite their enhanced protein flexibility, may be explained assuming that the exchange among compact and extended protein conformations occurs at a frequency that is higher with respect to the time scale of the proteolysis. Conversely, the increased conformational flexibility of the mutants probably perturbs the delicate equilibrium between structural rigidity and flexibility that must be assured to properly bind other proteins; this may account for the presence of some relevant post-translational effect of the mutations, i.e. a reduced affinity toward LARGE, resulting in a significant hypoglycosylation of the α-DG with loss of its functionality. Interestingly, we have recently reported that the mutation T190M reduces the mobility of α-DG within the membrane, as well as its clustering within the actin-rich domain, perturbing cell migration [56].

Although the mutants show remarkable differences in their conformations in solution, it is not easy to identify a correlation with different phenotypes featuring the related diseases. Indeed, the T192M mutation is associated to a severe form of limb-girdle muscular dystrophy with cognitive impairment, whereas the mutations V74I and D111N are related to a mild form of muscular dystrophy with asymptomatic hyperCKemia. Remarkably, the two diseases displayed apparently similar hypoglycosylated forms of α-DG. It can be hypothesized that such different phenotypes could be due to a different residual glycosylation of α-DG resulting in a differently reduced binding affinity towards its physiological partners that cannot be evaluated with the available analytical techniques. This is consistent with previous reports that pointed out the lack of correlation between the glycosylation levels of α-DG and clinical severity of the pathology [57]. A deeper insight into the biochemical mechanism underlining primary dystroglycanopathies could be achieved accumulating structural information on LARGE and on the way it interacts with α-DG-Nt in order to correctly glycosylate the α-DG mucin-like domain, a goal that we will hopefully reach in the next future.

## Supporting information

S1 Fig Thermal denaturation assay using DSF with SYPRO dye. Comparison of averaged unfolding fluorescent curves with the respective error bars for WT (A), V72I (B), D109N (C) and T190M (D). The fitting Boltzmann function limited to the temperature region of interest (first transition) is shown.
(TIF)

S2 Fig Guinier plots. Guinier plot representation of the SAXS data. The straight lines are the fitted data according to Guinier approximation to determine the radius of gyration and the scattering amplitude. A) V72I, B) D109N and C) T190M.
(TIF)

S3 Fig **Electrostatic potential maps of V72I (A) and WT (B).** The electrostatic potential (in *k*_*b*_T/*e*_*c*_ units) is mapped on solvent-accessible surface of the WT and V72I pathological mutant accessible surfaces. Negative potential is colored in blue, positive potential in red. Color scale varies between -2 and +2. Molecular models (stretch 105–116) are represented as ribbons with selected residues depicted as stick-and-ball.
(TIF)

S4 Fig **Experimental SAXS curves measured at different concentration:** A) V72I, B) D109N and C) T190M. The zoomed regions of these graphs at low angles are presented on the right column.
(TIF)

S5 Fig Comparison of the structural models of the WT and the three pathological mutants of α-DG-Nt. Typical CORAL models (green, red, blue and violet cartoon representations for the folded Ig-like and S6 domains, spheres for the restored missing fragments of WT (A), V72I (B), D109N (C) and T190M (D), respectively) overlaid to the respective CORAL models, where the domains have been fixed in the positions found in the crystal structures in yellow.
(TIF)

S6 Fig Distribution of the centers of masses of S6 domains: In the crystallographic models of the WT and the three mutants (represented by spheres) and in the respective CORAL models (represented by dots) respect to the Ig-like domain and its center of mass (represented by mesh). (TIF)

S1 Table Overall parameters calculated from SAXS experiments. SAXS data collection and processing information. Parameters derived from SAXS analysis are also reported.
(DOCX)

S2 Table Additional SAXS structural parameters: Radius of gyration (Rg), maximum dimension (Dmax), Porod volume (A^3^), and MW (Da). Dmax was obtained from the p(r) distribution using GNOM; I(0) (scattering intensity) was obtained from the scattering data by the Guinier analysis. Molecular mass (Mr) was estimated from comparison with I(0) intensity of the standard BSA sample.
(DOCX)

S3 Table Sequence alignments. Inter-specific alignments in the regions spanning the amino acid positions: A) 65–110 and B) 187–221, referring to the murine Ig-like domain of α-DG. Accession codes for dystroglycan sequences: *Homo sapiens* (q14118), *Macaca mulatta* (f6ru72), *Pan troglodytes* (h2qml8), *Gorilla gorilla gorilla* (g3r897), *Callitrix jacchus* (f6trx4), *Otolemur garnetii* (h0xc26), *Papio anubis* (a0a096mt62), *Chlorocebus sabaeus* (a0a0d9rr47), *Nomascus leucogenys* (g1r572), *Mus musculus* (q62165), *Rattus norvegicus* (f1m8k0), *Heterocephalus glaber* (a0a0p6jge5), *Oryctolagus cuniculus* (q28685), *Ictidomys tridecemlieatus* (i3n6c8), *Dipodomys ordii* (a0a1s3g6k3), *Felis catus* (b4xem8), *Canis lupus familiaris* (q9tsz6), *Sus scrofa* (q29243), *Bos tauros* (o18738), *Ovis aries* (w5pvz9), *Equus caballus* (f6x9u4), *Erinaceus europaeus* (a0a1s3wgq5), *Ailuropoda melanoleuca* (d2heu8), *Mustela putorius furo* (m3yqu5), *Loxodonta africana* (g3t6q9), *Sarcophilus harrisii* (g3wwr6), *Monodelphis domestica* (f7dbt8), *Gallus gallus* (a4var9), *Taeniopygia guttata* (h0z430), *Ficedula albicollis* (u3k2z0), *Anas platyrhynchos* (u3ibq2), *Meleagris gallopavo* (g1mw2), *Pelodiscus sinensis* (k7gfa6), *Anolis carolinensis* (g1kgb5), *Xenopus tropicalis* (f7ei21), *Xenopus laevis* (q7zx16), *Danio rerio* (q8jhu7), *Latimeria chalumnae* (h3b2q1), *Oreochromis niloticus* (i3kqf9), *Astyanax mexicanus* (w5l657), *Ictalurus punctatus* (w5ua28), *Aphyosemion striatum* (a0a1a7y3r0), *Nothobranchius furzeri* (a0a1a7zin9), *Nothobranchius rachovii* (a0a1a8plv8), *Nothobranchius kadleci* (a0a1a8dcc7), *Nothobranchius kuhntae* (a0a1a8jug2), *Nothobranchius pienaari* (a0a1a8ms21), *Nothobranchius kortausae* (a0a1a8hgb2), *Poeciliopsis prolifica* (a0a0s7hfk7), *Salmon salar* (a0a1s3p849).
(DOCX)
