# Supplementary figures and images for: The effect of the pathological V72I, D109N and T190M missense mutations on the molecular structure of α-dystroglycan
Source: PLoS One. 2017 Oct 16;12(10):e0186110. doi: 10.1371/journal.pone.0186110 (PMC5643065; doi:10.1371/journal.pone.0186110)

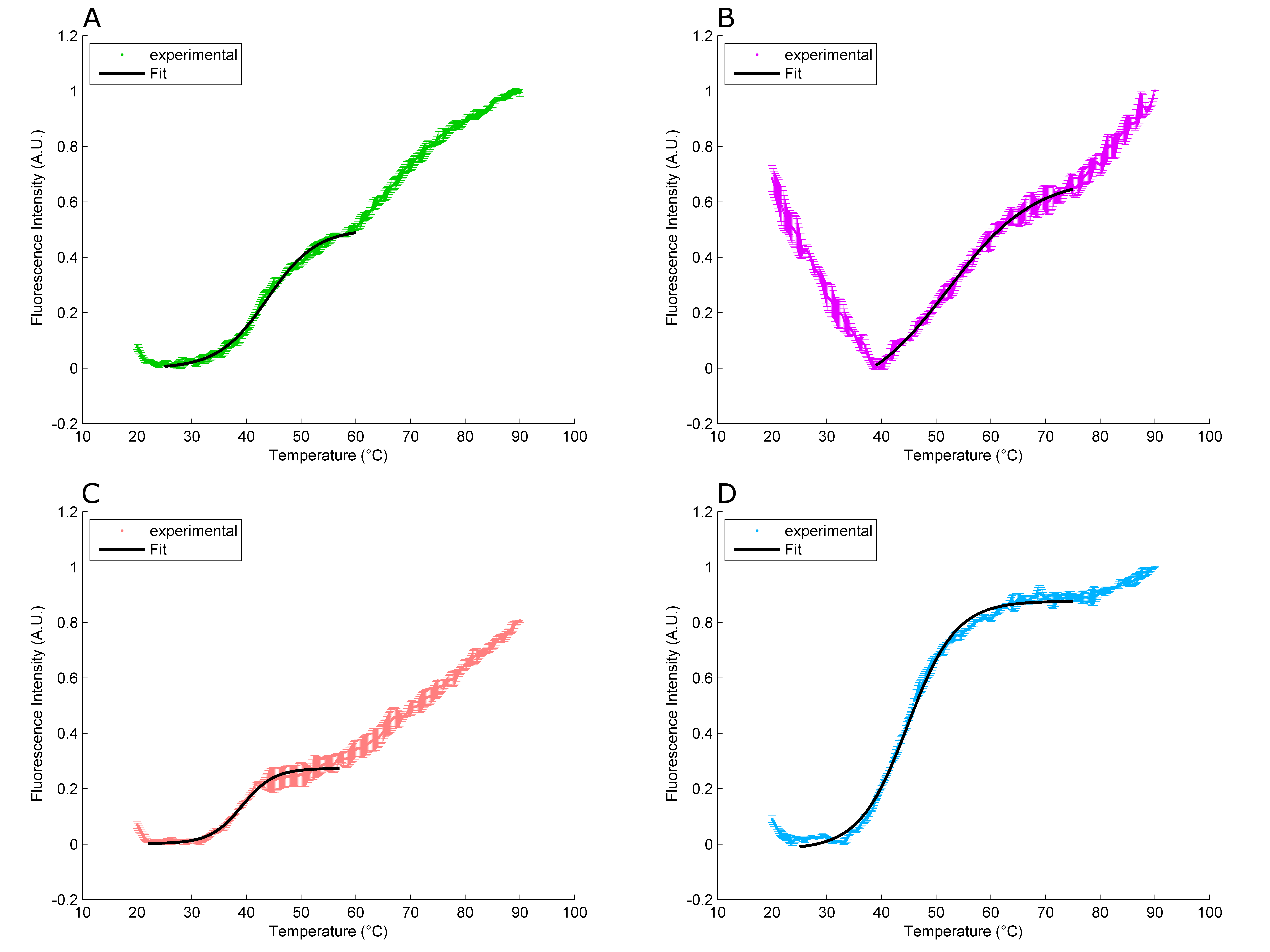

Supplement: S1 Fig — Comparison of averaged unfolding fluorescent curves with the respective error bars for WT (A), V72I (B), D109N (C) and T190M (D). The fitting Boltzmann function limited to the temperature region of interest (first transition) is shown. (TIF) [file pone.0186110.s001.tif]

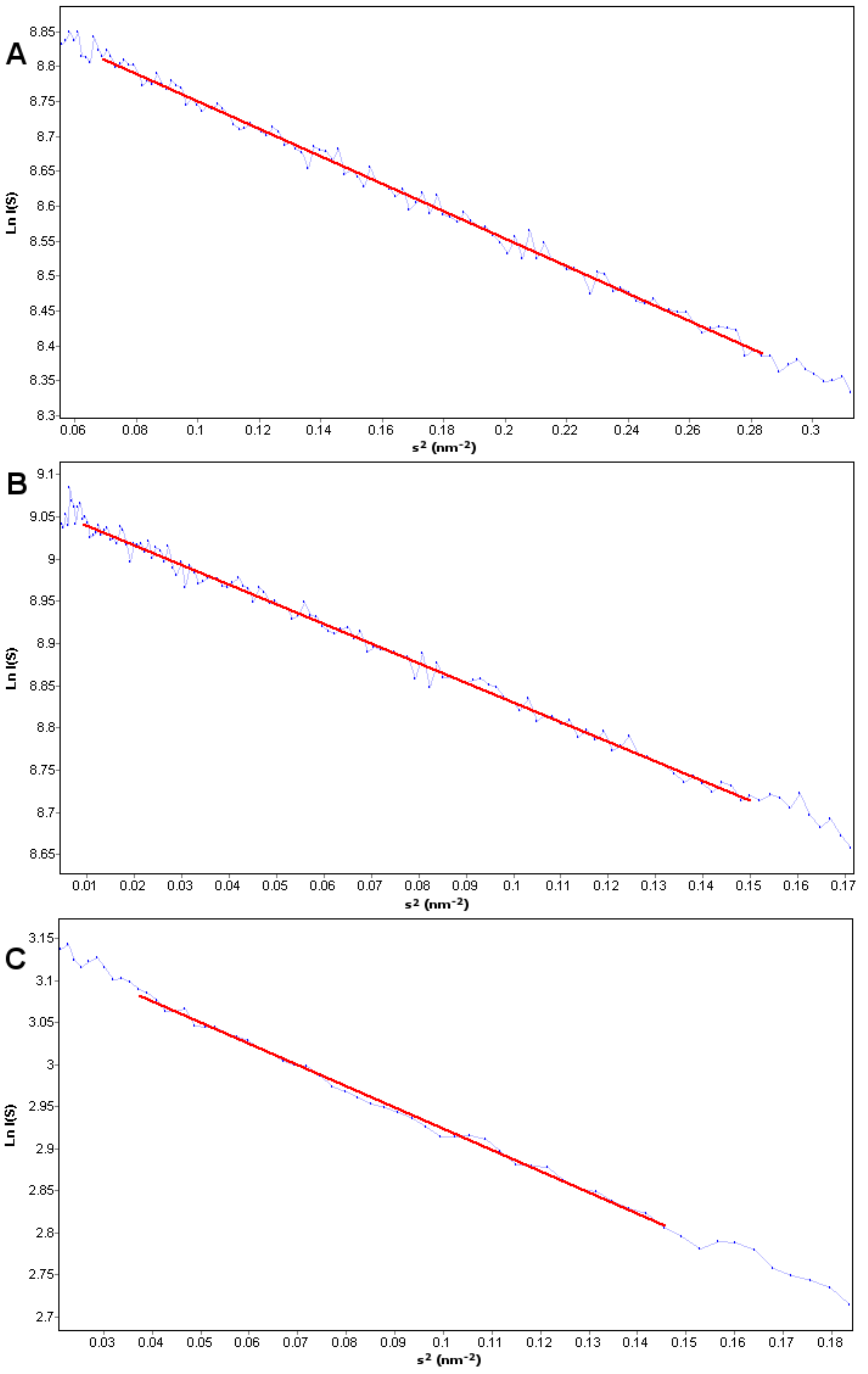

Supplement: S2 Fig — Guinier plot representation of the SAXS data. The straight lines are the fitted data according to Guinier approximation to determine the radius of gyration and the scattering amplitude. A) V72I, B) D109N and C) T190M. (TIF) [file pone.0186110.s002.tif]

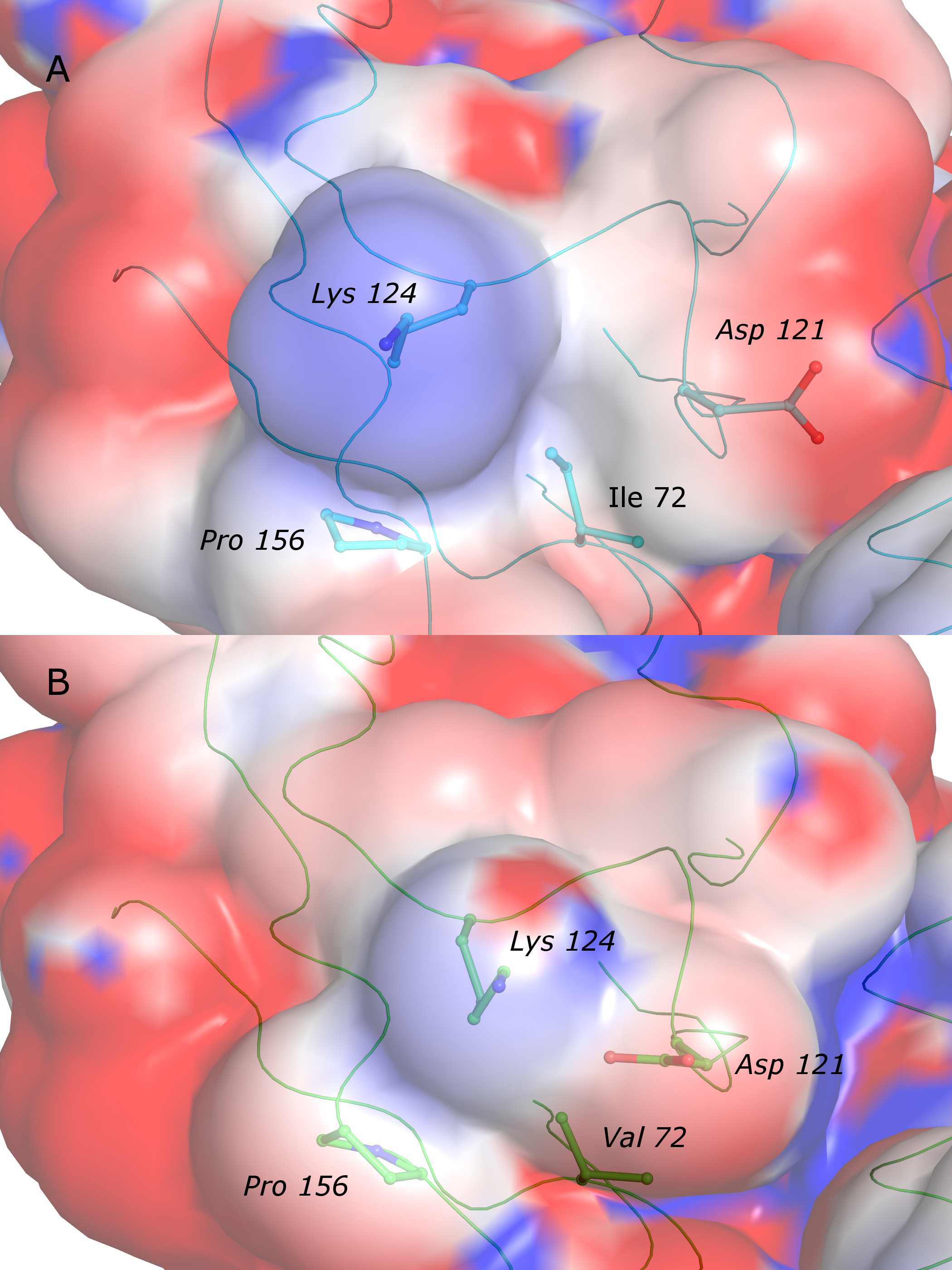

Supplement: S3 Fig — Electrostatic potential maps of V72I (A) and WT (B). The electrostatic potential (in kbT/ec units) is mapped on solvent-accessible surface of the WT and V72I pathological mutant accessible surfaces. Negative potential is colored in blue, positive potential in red. Color scale varies between -2 and +2. Molecular models (stretch 105–116) are represented as ribbons with selected residues depicted as stick-and-ball. (TIF) [file pone.0186110.s003.tif]

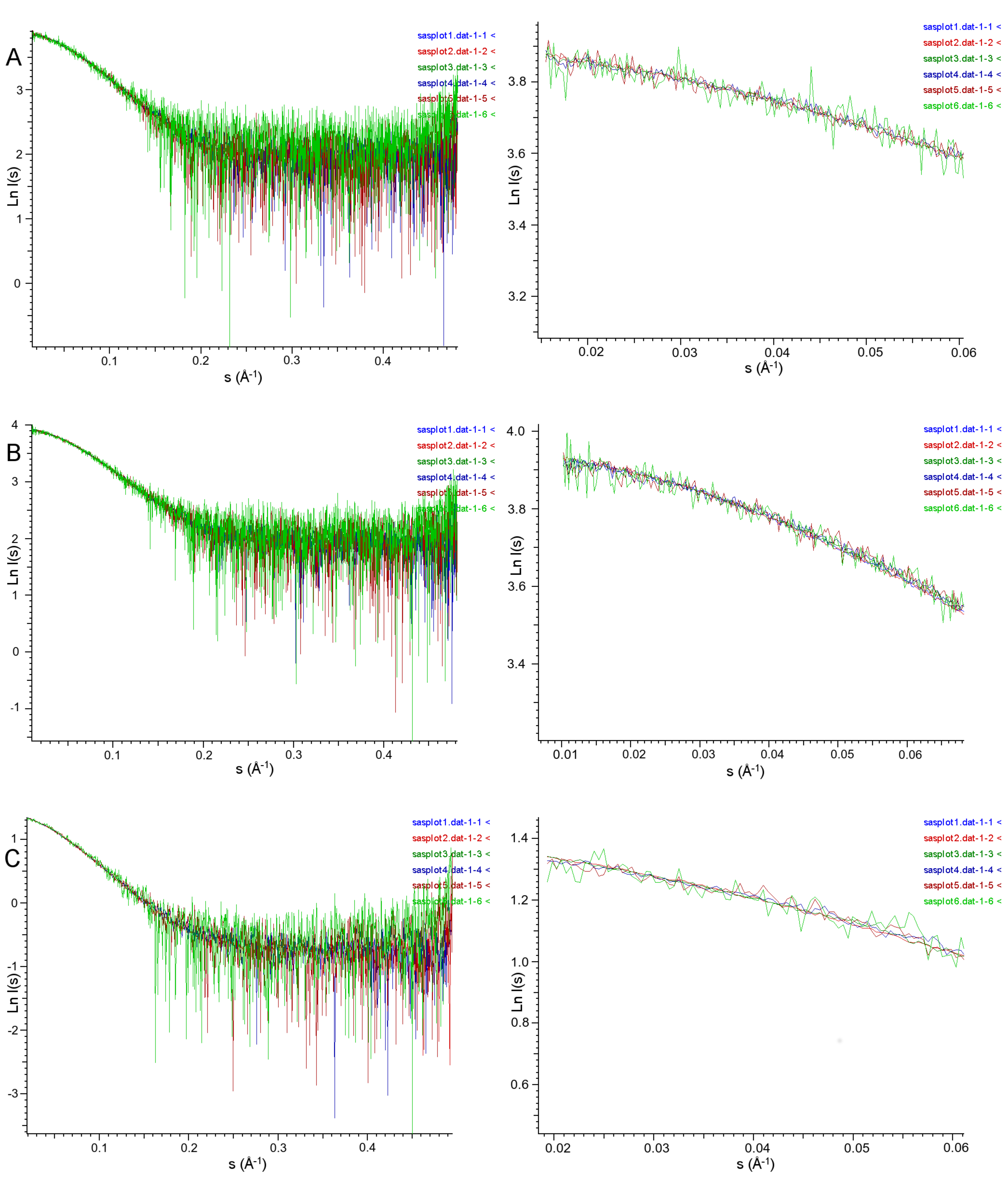

Supplement: S4 Fig — Experimental SAXS curves measured at different concentration: A) V72I, B) D109N and C) T190M. The zoomed regions of these graphs at low angles are presented on the right column. (TIF) [file pone.0186110.s004.tif]

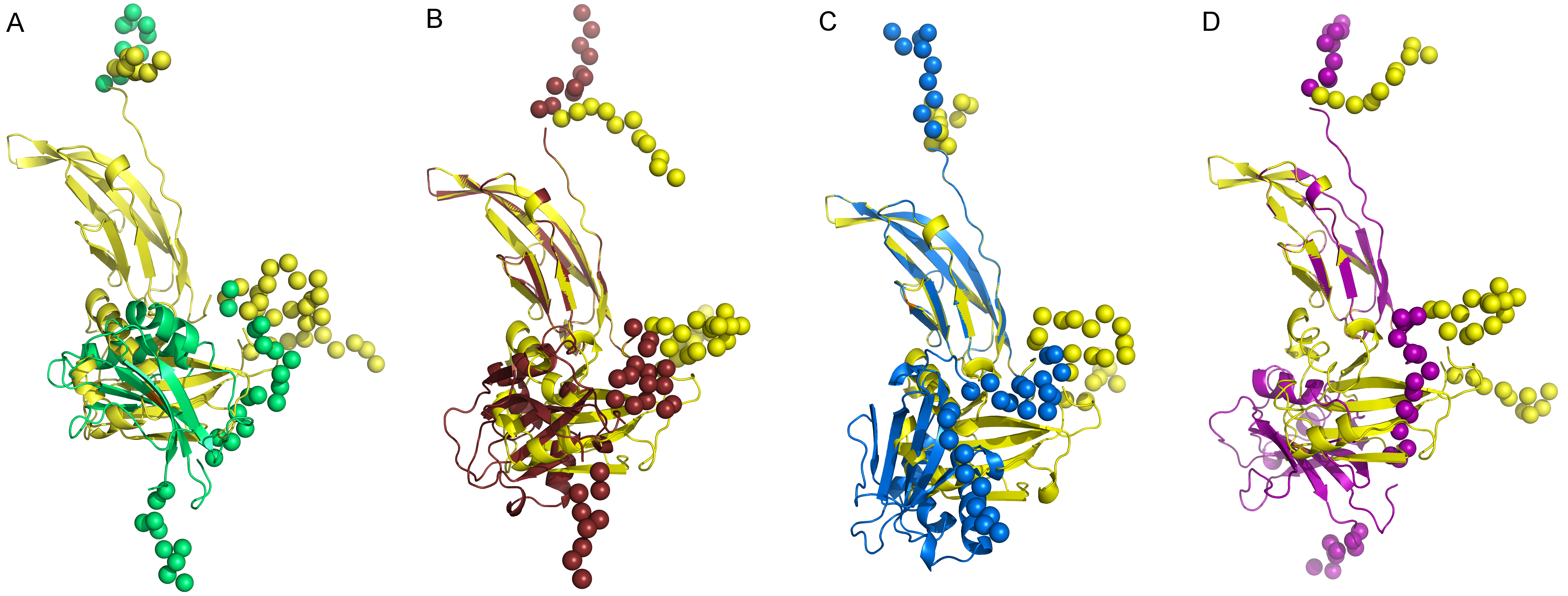

Supplement: S5 Fig — Typical CORAL models (green, red, blue and violet cartoon representations for the folded Ig-like and S6 domains, spheres for the restored missing fragments of WT (A), V72I (B), D109N (C) and T190M (D), respectively) overlaid to the respective CORAL models, where the domains have been fixed in the positions found in the crystal structures in yellow. (TIF) [file pone.0186110.s005.tif]

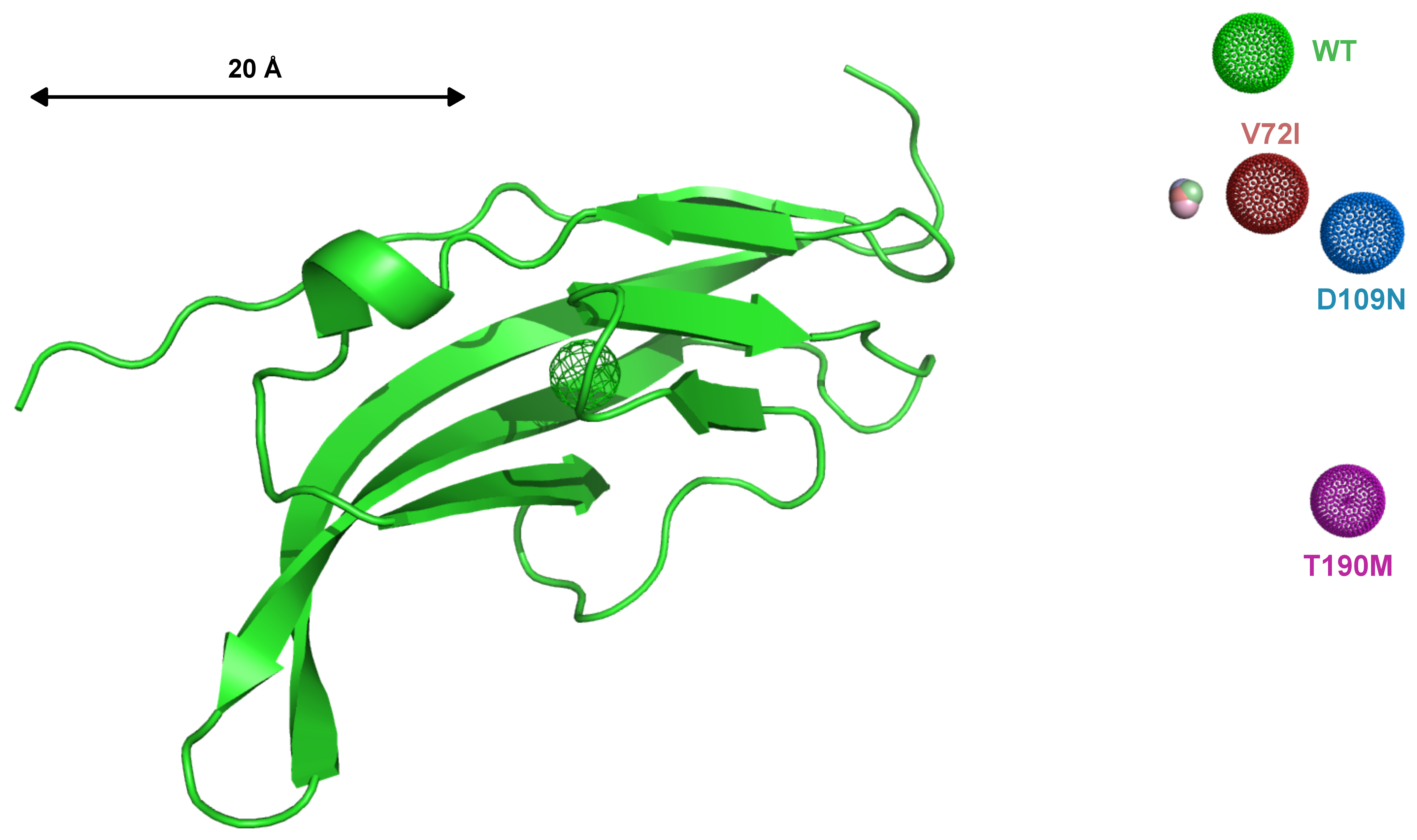

Supplement: S6 Fig — (TIF) [file pone.0186110.s006.tif]
