# Supplementary material for: The effect of the pathological V72I, D109N and T190M missense mutations on the molecular structure of α-dystroglycan
Source: PLoS One. 2017 Oct 16;12(10):e0186110. doi: 10.1371/journal.pone.0186110 (PMC5643065; doi:10.1371/journal.pone.0186110)
Supplement: S1 Table — SAXS data collection and processing information. Parameters derived from SAXS analysis are also reported. (DOCX) [file pone.0186110.s007.docx]

**S1 Table. Overall parameters calculated from SAXS experiments.** SAXS data collection and processing information. Parameters derived from SAXS analysis are also reported.

| sample | R_g_ (Å) | D_max_ (Å) | V_p_ (Å^3^) | MM_exp_ (kDa) | Χ_crystal_ | Χ_CORAL_ | Χ_ab-initio_ | Χ_EOM_ |
| --- | --- | --- | --- | --- | --- | --- | --- | --- |
| WT | 25.20±0.04 | 90±3 | 44000±2000 | 25.1±3.0 | 2.87 | 0.84 | 0.81 | 0.63 |
| V72I | 24.20±0.04 | 80±2 | 43000±2000 | 27.5±3.0 | 1.69 | 1.00 | 1.21 | 0.98 |
| D109N | 26.40±0.04 | 95±3 | 47000±2000 | 29.5±3.0 | 8.55 | 1.18 | 1.31 | 1.17 |
| T190M | 27.20±0.04 | 100±3 | 51000±3000 | 23.3±3.0 | 15.07 | 1.17 | 1.04 | 1.32 |
