# Supplementary material for: The effect of the pathological V72I, D109N and T190M missense mutations on the molecular structure of α-dystroglycan
Source: PLoS One. 2017 Oct 16;12(10):e0186110. doi: 10.1371/journal.pone.0186110 (PMC5643065; doi:10.1371/journal.pone.0186110)
Supplement: S2 Table — Dmax was obtained from the p(r) distribution using GNOM; I(0) (scattering intensity) was obtained from the scattering data by the Guinier analysis. Molecular mass (Mr) was estimated from comparison with I(0) intensity of the standard BSA sample. (DOCX) [file pone.0186110.s008.docx]

**S2 Table. Additional SAXS structural parameters**: radius of gyration (Rg), maximum dimension (Dmax), Porod volume (A^3^), and MW (Da). Dmax was obtained from the p(r) distribution using GNOM; I(0) (scattering intensity) was obtained from the scattering data by the Guinier analysis. Molecular mass (Mr) was estimated from comparison with I(0) intensity of the standard BSA sample.

| **Data collection parameters** | **V72I** | **D109N** | **T190M** |
| --- | --- | --- | --- |
| Instrument | P12 (PETRA III) | P12 (PETRA III) | BM29 (ESRF) |
| Beam geometry (mm^2^) | 0.2 x 0.12 | 0.2 x 0.12 | 0.5x0.5 |
| Wavelength (Å) | 1.24 | 1.24 | 0.99 |
| s range (Å^-1^) | 0.003–0.445 | 0.003–0.445 | 0.0025–0.6 |
| Concentration range (mg/ mL) | 0.12-5.29 | 0.18-6.60 | 0.22-3.74 |
| Temperature (K) | 283 | 283 | 283 |
| **Structural parameters** | | | |
| I(0) (A.U.) [from p(r)] | 7792±16 | 8579±18 | 23.94±0.04 |
| Rg (A) [from p(r)] | 24.40±0.04 | 26.40±0.04 | 27.90±0.04 |
| I(0) (A.U.) [from Guinier] | 7796±16.7 | 8622,9±18,2 | 23.83±0.04 |
| Rg (A) [from Guinier] | 24.20±0.04 | 26.40±0.04 | 27.20±0.04 |
| Dmax (Å) | 80±2 | 95±3 | 100±3 |
| Porod volume estimate (Å^3^) | 43000±2000 | 47000±2000 | 51000±3000 |
| **Molecular mass determination (Da)** | | | |
| Molecular mass MM [from I(0)] | 27500±3000 | 29500±3000 | 23300±3000 |
| Molecular mass MM [from Porod volume] | 25300±3000 | 27600±3000 | 30000±3000 |
| Calculated monomeric MM from sequence | 28500 | 28500 | 28500 |
| **Software employed** | | | |
| Primary data reduction | PRIMUS | PRIMUS | PRIMUS |
| Data processing | GNOM | GNOM | GNOM |
| Ab initio analysis | DAMMIN | DAMMIN | DAMMIN |
| Validation and averaging | DAMAVER | DAMAVER | DAMAVER |
| Rigid body modeling | CORAL/EOM | CORAL/EOM | CORAL/EOM |
| Computation of model intensities | CRYSOL | CRYSOL | CRYSOL |
| Three-dimensional graphic representations | PYMOL | PYMOL | PYMOL |
