# Supplementary material for: The effect of the pathological V72I, D109N and T190M missense mutations on the molecular structure of α-dystroglycan
Source: PLoS One. 2017 Oct 16;12(10):e0186110. doi: 10.1371/journal.pone.0186110 (PMC5643065; doi:10.1371/journal.pone.0186110)
Supplement: S3 Table — Inter-specific alignments in the regions spanning the amino acid positions: A) 65–110 and B) 187–221, referring to the murine Ig-like domain of α-DG. Accession codes for dystroglycan sequences: Homo sapiens (q14118), Macaca mulatta (f6ru72), Pan troglodytes (h2qml8), Gorilla gorilla gorilla (g3r897), Callitrix jacchus (f6trx4), Otolemur garnetii (h0xc26), Papio anubis (a0a096mt62), Chlorocebus sabaeus (a0a0d9rr47), Nomascus leucogenys (g1r572), Mus musculus (q62165), Rattus norvegicus (f1m8k0), Heterocephalus glaber (a0a0p6jge5), Oryctolagus cuniculus (q28685), Ictidomys tridecemlieatus (i3n6c8), Dipodomys ordii (a0a1s3g6k3), Felis catus (b4xem8), Canis lupus familiaris (q9tsz6), Sus scrofa (q29243), Bos tauros (o18738), Ovis aries (w5pvz9), Equus caballus (f6x9u4), Erinaceus europaeus (a0a1s3wgq5), Ailuropoda melanoleuca (d2heu8), Mustela putorius furo (m3yqu5), Loxodonta africana (g3t6q9), Sarcophilus harrisii (g3wwr6), Monodelphis domestica (f7dbt8), Gallus gallus (a4var9), Taeniopygia guttata (h0z430), Ficedula albicollis (u3k2z0), Anas platyrhynchos (u3ibq2), Meleagris gallopavo (g1mw2), Pelodiscus sinensis (k7gfa6), Anolis carolinensis (g1kgb5), Xenopus tropicalis (f7ei21), Xenopus laevis (q7zx16), Danio rerio (q8jhu7), Latimeria chalumnae (h3b2q1), Oreochromis niloticus (i3kqf9), Astyanax mexicanus (w5l657), Ictalurus punctatus (w5ua28), Aphyosemion striatum (a0a1a7y3r0), Nothobranchius furzeri (a0a1a7zin9), Nothobranchius rachovii (a0a1a8plv8), Nothobranchius kadleci (a0a1a8dcc7), Nothobranchius kuhntae (a0a1a8jug2), Nothobranchius pienaari (a0a1a8ms21), Nothobranchius kortausae (a0a1a8hgb2), Poeciliopsis prolifica (a0a0s7hfk7), Salmon salar (a0a1s3p849). (DOCX) [file pone.0186110.s009.docx]

**S3 Table. Sequence Alignments.**

Inter-specific alignments in the regions spanning the amino acid positions: A) 65-110 and B) 187-221, referring to the murine Ig-like domain of α-DG. Accession codes for dystroglycan sequences: *Homo sapiens* (q14118), *Macaca mulatta* (f6ru72), *Pan troglodytes* (h2qml8), *Gorilla gorilla gorilla* (g3r897), *Callitrix jacchus* (f6trx4), *Otolemur garnetii* (h0xc26), *Papio anubis* (a0a096mt62), *Chlorocebus sabaeus* (a0a0d9rr47), *Nomascus leucogenys* (g1r572), *Mus musculus* (q62165), *Rattus norvegicus* (f1m8k0), *Heterocephalus glaber* (a0a0p6jge5), *Oryctolagus cuniculus* (q28685), *Ictidomys tridecemlieatus* (i3n6c8), *Dipodomys ordii* (a0a1s3g6k3), *Felis catus* (b4xem8), *Canis lupus familiaris* (q9tsz6), *Sus scrofa* (q29243), *Bos tauros* (o18738), *Ovis aries* (w5pvz9), *Equus caballus* (f6x9u4), *Erinaceus europaeus* (a0a1s3wgq5), *Ailuropoda melanoleuca* (d2heu8), *Mustela putorius furo* (m3yqu5), *Loxodonta africana* (g3t6q9), *Sarcophilus* *harrisii* (g3wwr6), *Monodelphis domestica* (f7dbt8), *Gallus gallus* (a4var9), *Taeniopygia guttata* (h0z430), *Ficedula albicollis* (u3k2z0), *Anas platyrhynchos* (u3ibq2), *Meleagris gallopavo* (g1mw2), *Pelodiscus sinensis* (k7gfa6), *Anolis carolinensis* (g1kgb5), *Xenopus tropicalis* (f7ei21), *Xenopus laevis* (q7zx16), *Danio rerio* (q8jhu7), *Latimeria chalumnae* (h3b2q1), *Oreochromis niloticus* (i3kqf9), *Astyanax mexicanus* (w5l657), *Ictalurus punctatus* (w5ua28), *Aphyosemion striatum* (a0a1a7y3r0), *Nothobranchius furzeri* (a0a1a7zin9), *Nothobranchius rachovii* (a0a1a8plv8), *Nothobranchius kadleci* (a0a1a8dcc7), *Nothobranchius kuhntae* (a0a1a8jug2), *Nothobranchius pienaari* (a0a1a8ms21), *Nothobranchius kortausae* (a0a1a8hgb2), *Poeciliopsis prolifica* (a0a0s7hfk7), *Salmon salar* (a0a1s3p849).

***Homo sapiens*           67 PDGTAVVGRSFRVTIPTDLIAS-SGDIIKVSAAGKEALPSWLHWDS 112**

***Macaca mulatta*         66 PDGTAVVGRSFRVTIPTDLIAS-SGDIIKVSAAGKEALPSWLHWDP 111**

***Pan troglodytes*         67 PDGTAVVGRSFRVTIPTDLIAS-SGDIIKVSAAGKEALPSWLHWDS 112**

***Gorilla gorilla*     67 PDGTAVVGRSFRVTIPTDLIAS-SGDIIKVSAAGKEALPSWLHWDS 112**

***Callitrix jacchus*       67 PDGTAVVGRSFRVTIPTDLIAS-SGDIIKVSAAGKEALPSWLHWDP 112**

***Otolemur garnetii*         67 PDGTAVVGRSFRVTIPTDVIAS-NGEIIKVSAVGKEALPSWLYWDL 112**

***Papio anubis*     66 PDGTAVVGRSFRVTIPTDLIAS-SGDIIKVSAAGKEALPSWLHWDP 111**

***Chlorocebus sabaeus*      66 PDGTAVVGRSFRVTIPTDLIAS-SGDIIKVSAAGKEALPSWLHWDP 111**

***Nomascus leucogenys*        67 PDGTAVVGRSFRVTIPTDLIAS-SGDIIKVSAAGKEALPSWLHWDS 112**

***Mus musculus*           65 PDGTAVVGRSFRVSIPTDLIAS-SGEIIKVSAAGKEALPSWLHWDP 110**

***Rattus norvegicus* 65 PDGTAVVGRSFRVSIPTDLIAS-SGEIIKVSAAGKEALPSWLHWDP 110**

***Heterocephalus glaber*      65 PDGTAVVGRLFRVTVPTDLITS-SGEIIKVSAAGKEALPSWLHWDP 120**

***Oryctolagus cuniculus* 67 PDGTAVVGRSFRVTIPTDLIGS-SGEVIKVSTAGKEVLPSWLHWDP 112**

***Ictidomys tridecemlieatus*  65 PDGTAIVGRSFQVTIPTDLIAS-SGEIIKVSAAGKEALPSWLHWDP 110**

***Dipodomys ordii*     67 PDGTAVVGRSFRVTIPTDLIAS-SGEIIKVSTAGKEALPSWLHWDP 112**

***Felis catus*       64 PDGTAVVGRSFRVTIPMDLIAS-NGELIKVSTAGKEALPSWLHWDP 109**

***Canis lupus familiaris*     64 PDGIAVVGRSFRVTIPMDLIAS-NGELVKVSAVGKEVLPSWLHWDP 109**

***Sus scrofa*             67 PDGTAVVGRSFRVTIPTDLIAS-GGEIIKVSAAGKEALPSWLHWDP 112**

***Bos tauros*           67 PDGTAVVGRSFRVTIPTDLIAS-NGEVIKVSAAGKEALPSWLHWDP 112**

***Ovis aries*   67 PDGTAVVGRSFRVTIPTDLIAS-NGEVIKVSAAGKEALPSWLHWDP 112**

***Equus caballus* 67 PDGTAVVGRSFRVTIPTDLIAS-NGEVIKVSAAGKEALPSWLHWDS 112**

***Erinaceus europaeus*     67 PDGTAVVGRSFRVTIPTDLISS-NGEVIKVSAAGKEALPSWLHWDP 112**

***Ailuropoda melanoleuca* 64 PDGTAVVGRSFRVTIPTDLIAS-SGELIKVSAAGKEALPSWLHWDP 109**

***Mustela putorius furo*      64 PDGTAVVGRSFRVTIPADSVAS-NGELIKVSTAGKEALPSWLHWDP 109**

***Loxodonta africana*         65 PDGMAVVGRSFRVTIPTDLIAS-SGEVIKVSAAGKETLPSWLHWDP 120**

***Sarcophilus harrisii*    67 PDGIAVVGRSFRVAIPTDLIAS-NGEIIKMSEAGKEVLPSWLHWDP 112**

***Monodelphis domestica*      68 PDGIAVVGRSFRVAIPTDLIAS-NGEIIKMSEAGKEVLPSWLHWDP 113**

***Gallus gallus*    66 PDSSAVVGRFFRVSIPTDLIAS-NGEAVQVSEAGKESLPSWLHWNA 111**

***Taeniopygia guttata*        67 PDSSAVVGRYFRVSIPTDLIAS-NGEVVQISEAGKDSLPSWLHWNA 112**

***Ficedula albicollis*        67 PDSSAVVGRYFRASIPTELIAA-NGEAVQISEAGKDSLPSWLHWNA 112**

***Anas platyrhynchos*         69 PDSSAVVGRFFRVSIPTDLIAS-NGEVVQISEAGKESLPSWLHWNA 114**

***Meleagris gallopavo*        66 PDSSAVVGRFFRVSIPTDLIAS-NGEVVQVSEAGKESLPSWLHWNA 111**

***Pelodiscus sinensis*     66 PDSSAVVGRSFRVLIPADVIAS-SGEMVQISEVGKDSLPSWLHWEP 111**

***Anolis carolinensis*        65 PDSSAVVGRSFRVTIPTDLIAS-NGEVIQIIEAGKESLPSWLHWES 120**

***Xenopus tropicalis*         67 PDSSAVVGRPFKIHIPTEFLAS-SGETIKIFEVGKEILPSWLHWEA 112**

***Xenopus laevis*           65 PDSSALVGRPFKIHIPTEFLAS-SGETIKISEVGKETLPSWLHWEG 120**

***Danio rerio*         80 PDSSAVVGRVFRLQVPIKAKDS--GSIVKITEASKDVLPAWLHWDA 124**

***Poeciliopsis prolifica* 107 PDCSAIVGQVFQLKVPPGPDHA--SCNVQLTEMGRETLPSWLYWDK 157**

***Salmon salar*     100 PDSSAVVGRVFQMKVPVKTDY---TNNTKITESGKETLPAWLHWDW 142**

***Oreochromis niloticus*   108 PDTSAMVGCIFQMKVPNKMEDVYLGDIIKITEMGKDSLPEWLHWDA 153**

***Astyanax mexicanus*         62 SDTVAVVGQMFWMRIPLPSADCHSTD--IFSEVGSAPFPSWLYWDN 106**

***Ictalurus punctatus*        83 PDSSAVVGRMFQMQIPTKAKDS--GSIVKITEAGKDTLPSWLHWEP 127**

***Aphyosemion striatum*     107 PGTLAVVGRIFQIKVPNKMEDVYLGDI-KVSEMGKDSLPSWLHWDA 151**

***Nothobranchius furzeri*    114 PDTSAVVGRIFQIKVPNRMEDVYLGDIIKVSEMGKDSLPSWLHWDA 159**

***Nothobranchius rachovii*    89 PDSSAIVGQVFQLKVPLRPTHE--SCSVHLTEMGKKTLPSWLYWDK 132**

***Nothobranchius kadleci*   107 PDTSAVVGRIFQIKVPNRMEDVYLGDIIKVSEMGKDSLPSWLHWDA 159**

***Nothobranchius kuhntae*    107 PDTSAVVGRIFQIKMPNRMEDVYLGDIIKVSELGKESLPSWLHWDA 159**

***Nothobranchius kortausae* 107 PDTSAVVGRIFQIKVPNRMEDVYLGDIIKVSEMGKDSLPSWLHWDA 159**

***Nothobranchius pienaari*   107 PDTSAVVGRIFQIKVPNRMEDVYLGDIIKVSEMGKDSLPSWLHWVA 139**

***Latimeria chalumnae*       66 PDKSAVVGRVFQMTIPSEMLSV-NGQQIKVTEAGKESLPSWLHWKP 111**

* ** * :* . . :* ** :

**S3A Table.**

***Homo sapiens*           189 TVLTVILDADLTKMTPKQRIDLLHRMRSFSEVELH  223**

***Macaca mulatta*         188 TVLTVILDADLTKMTPKQRIDLLHRMRSFSEVELH  222**

***Pan troglodytes*         189 TVLTVILDADLTKMTPKQRIDLLHRMRSFSEVELH  223**

***Gorilla gorilla*    189 TVLTVILDADLTKMTPKQRIDLLHRMRSFSEVELH  223**

***Callitrix jacchus*       189 TVLTVILDADLTKMTPKQRIDLLHRMRSFSEVELH  223**

***Otolemur garnetii*         189 TVLTVILDADLTKMTPKQRVDLLHKMRRFSEVELH  223**

***Papio anubis*     188 TVLTVILDADLTKMTPKQRIDLLHRMRSFSEVELH  222**

***Chlorocebus sabaeus*     189 TVLTVILDADLTKMTPKQRIDLLHRMRSFSEVELH  222**

***Nomascus leucogenys*       189 TVLTVILDADLTKMAPKQRIDLLHRMRSFSEVELH  223**

***Mus musculus*           187 TVLTVILDADLTKMTPKQRIDLLNRMQSFSEVELH  221**

***Rattus norvegicus* 187 TVLTVILDADLTKMTPKQRIDLLNRMQSFSEVELN  221**

***Heterocephalus glaber*     187 TVLTVILDADLTKMTPKQRIDLLHRMQSFSEVEIH  221**

***Oryctolagus cuniculus* 189 TVLTVILDADLTKMTPKQRIDLLHRMQSFSEVELH  223**

***Ictidomys tridecemlieatus* 187 TVLTVILDADLTKMTPKQRIDLLHKMQSFSEVELH  221**

***Dipodomys ordii*     189 TVLTVILDADLTKMTPKQRLDLLHRMRSFSEVELH  222**

***Felis catus*       186 TVLTVILDADLTKMTPKQRIDLLHRMQSFSEVELH  220**

***Canis lupus familiaris*    188 TVLTVILDADLTKMTPKQRIDLLHRMRSFSEVELH  220**

***Sus scrofa*             189 TVLTVILDADLTKMIPKQRLDLLQRMQSFSEVELH  223**

***Bos tauros*           189 TVLTVILDADLTKMTPKQRIDLLRRMRGFSEVEPH  223**

***Ovis aries*   189 TVLTVILDADLTKMTPKQRIDLLRRMRGFSEVEPH  223**

***Equus caballus* 189 TVLTVILDADLTKMTPKQRIDLLHRMRSFSEVELH  223**

***Erinaceus europaeus*    189 TVLTVILDADLTKMTPKERLDLLHRMQSFSQVELH  223**

***Ailuropoda melanoleuca* 186 TVLTVILDADLTKMTPKQRIELLHRMRSFSEVELP  220**

***Mustela putorius furo*     186 TVLTVILDADLTKMTPKQRLDLLHRMRSFSEVELS  220**

***Loxodonta africana*        187 TVLTVILDADLTKMTPKQRIDLLHSMQSFSGVELH  221**

***Sarcophilus harrisii*    189 TILTVILDADLTKMTPKQRVELLNRMKSFSEVELH  223**

***Monodelphis domestica*     190 TILTVILDADLTKMTPKQRVELLNRMKRFSEVELH  224**

***Gallus gallus*   188 TILTVILDADLTKMTPKQRIELLNRMRSFSEVELH  222**

***Taeniopygia guttata*       189 TILTVILDADLTKMTPKQRIELLNRMRSFSEVELH  223**

***Ficedula albicollis*       189 TILTVILDADLTKMTPKQRIELLNRMRSFSEVELH  223**

***Anas platyrhynchos*        191 TVLTVILDADLTKMTPKQRIELLNRMRSFSEVELH  225**

***Meleagris gallopavo*       188 TILTVILDADLTKMTPKQRIELLNRMRSFSEVELH  222**

***Pelodiscus sinensis*     188 TILTVILDADLTKMTPKQRVELVTRMRRFSEVELH  222**

***Anolis carolinensis*       187 TILTVILDADLTKMTPKQRIELLNRMRTFSEVELY  221**

***Xenopus tropicalis*       187 TILTVILDADLTKMTPKQRVDLLNRMRDFSEVELY  221**

***Xenopus laevis*          185 TLLTVILDADLTKMTPKQRVDLLNRMRDFSEVELF  219**

***Danio rerio*         199 TVLTVILDADLTKMSSKQRVELLAKMKKFSGMGLQ  233**

***Latimeria chalumnae*       188 TILTVILDADLIKMTPKQRVELLSRMRKFSEVELH  222**

***Oreochromis niloticus*   229 TVLTVILDADLTKMSSEQRVELLDNMRSFSGVGLQ  263**

***Astyanax mexicanus*        201 TVLTIILDADLIKMNSRERLALLQKMSHFASVPPE  235**

***Ictalurus punctatus*       201 TVLTVILDADLTKMSSKQRVELLASMKKFSGVGLQ  235**

***Aphyosemion striatum*     226 TVLTVILDADLTKMSSEQRVELVDRMRSFSGVDLQ  260**

***Nothobranchius furzeri*    234 TILTVILDADLTKMSSEQRVELLDHMRSFSGVDLQ  268**

***Nothobranchius rachovii*   210 TVLTVILDADLTKMFAEQRVILMEVMRKFSHVPLE  244**

***Nothobranchius kadleci*   234 TILTVILDADLTKMSSEQRVELLDHMRSFSGVDLQ  268**

***Nothobranchius kuhntae*    234 TILTVILDADLTKMSSEQRVELLDHMRSFSGVDLQ  268**

***Nothobranchius pienaari*   234 TILTVILDADLTKMSSEQRVELLDHMRSFSGVDLQ  268**

***Nothobranchius kortausae* 234 TILTVILDADLTKMSSEQRVELLDHMRSFSGVDLQ  268**

***Poeciliopsis prolifica* 235 TVLTVILDADLTKMVAEQRVSLLGLMRKFSHVPVE  269**

***Salmon salar*     210 TVLTVILDADLTKMSSRQRVELLSKMKKFSGVALQ  244**

***::*::** : : :: : : * :**

**S3B Table.**
